# Unraveling the physiological and ultrastructural responses of wheat to combat cobalt stress and the protective role of *Jania rubens* related to antioxidant defense and cellular integrity

**DOI:** 10.3389/fpls.2025.1621482

**Published:** 2025-07-04

**Authors:** Gehad A. Ragab, Afaf A. Nessem, Mostafa E. Elshobary, Joachim Henjes, Esraa O. Razzaky

**Affiliations:** ^1^ Department of Botany and Microbiology, Faculty of Science, Tanta University, Tanta, Egypt; ^2^ Aquaculture Research, AWI—Helmholtz Centre for Polar and Marine Research, Bremerhaven, Germany

**Keywords:** antioxidants, cobalt stress, seaweeds, ultrastructure, biostimulant

## Abstract

Cobalt (Co), while beneficial in trace amounts for biological systems, can severely impact plant growth at elevated levels in contaminated soils. This study investigated the physiological, biochemical and subcellular effects of Co toxicity on wheat (*Triticum aestivum* L.) and evaluated, for the first time, the protective potential of *Jania rubens* extract. The algal extract analysis demonstrated its rich content of amino acids, minerals, phytohormones, and fatty acids. Wheat seedlings were subjected to cobalt chloride (150 mM) irrigation, which was previously primed with either water or *J. rubens* extract. Co stress significantly impaired growth by reducing water content and essential nutrients (K, Mg, and Fe), leading to a 42.42 and 23.8% decrease, respectively, in root and shoot biomasses, a 9% reduction in photosynthetic efficiency, visible chlorosis, and root thickening. Stress exposure also induced oxidative damage, shown by 67.1% increase in hydrogen peroxide and a 170.1% rise in malondialdehyde content, accompanied by membrane leakage and reduced antioxidant enzyme activities. Ultrastructural analysis confirmed morphophysiological and biochemical disruptions at the cellular level. Priming with *J. rubens* extract significantly alleviated these effects by enhancing nutrient uptake, increasing root and shoot biomasses by 78.94% and 58.33%, respectively, reducing oxidative damage and maintaining cellular homeostasis. It also preserved chloroplast structure, nucleus, and cell wall microtubules, maintaining overall cellular integrity and antioxidant efficiency. Our findings demonstrate that *Jania rubens* extract offers a promising and novel biogenic strategy for enhancing wheat resilience to cobalt contamination through its nutritional and antioxidant properties.

## 1 Introduction

Environmental pollution with heavy metals has become alarmingly serious due to rapid urbanization and unlimited industrialization. Although cobalt (Co) is not an essential element for plants, it may support certain enzymatic activities in some species (Salam et al., 2024). This trace element can eventually seep into the soil due to mining and smelting activities, agricultural additives, and metal refineries (Zaborowska et al., 2016). However, excessive cobalt exposure disrupts critical metabolic processes in plants (Anjum et al., 2015; Mahey et al., 2020). At elevated concentrations, cobalt (Co) exerts deleterious effects on plant growth, impairs chlorophyll biosynthesis and cell division, and disrupts water and nutrient uptake and translocation, primarily through the induction of oxidative stress (Akeel and Jahan, 2020). Despite its high toxicity, relatively few studies have investigated cobalt accumulation and defense responses in vascular plants. Thus, a deeper understanding of Co’s phytotoxicity is essential to mitigate its threats to crop productivity and human health.

Biotechnological innovations now offer eco-friendly solutions for enhancing crop resilience under environmental stress. In this context, biologically based fertilizers reduce dependency on synthetic inputs and lower environmental risks in agroecosystems. Marine macroalgae (seaweeds) are regarded as a potential resource for bioactive compounds such as polysaccharides, proteins, amino acids, peptides, fatty acids, minerals, vitamins, and plant growth regulators (Hamed et al., 2018; Hussein et al., 2021; Ashour et al., 2023). Various aqueous algal extracts were prepared through methods such as boiling, autoclaving, or homogenization and exhibited beneficial impacts on the health, growth, and production of crop plants (Hamed et al., 2018; Ashour et al., 2021). Both seed presoaking and foliar applications of aqueous algal extracts can be utilized as plant biostimulators (Vasantharaja et al., 2019; El-Shenody et al., 2023). Besides, seaweed-derived organic fertilizers can also improve tolerance for both biotic and abiotic stresses (Yadav et al., 2016; Hussein et al., 2021). However, the prospective mode of action of algal extracts concerning both growth-promoting and alleviating activity is still not fully explored as a bio-safe and effective plant application.

*Jania rubens* is naturally abundant along the Mediterranean coast of Egypt, especially in areas like Abu Qir Bay and the Eastern Harbor in Alexandria. Multiple studies confirm its regular seasonal presence and accessibility in these regions, where it has been collected for biochemical and biostimulant analysis (Khairy and El-Sheikh, 2015; Ismail et al., 2023). This local availability makes it a practical and sustainable source for biostimulant applications in Egyptian agriculture. *J. rubens*, a benthal red marine macroalga belonging to the *Corallinaceae* family. It is heavily infused with calcium carbonate, in the form of calcite, via the bio-mineralization phenomenon (Ismail et al., 2023). Further, *J. rubens* is an abundant source of key micro - and macro-nutrients, antioxidants, and bioactive ingredients (Dixit and Reddy, 2017). *J. rubens* was selected over other seaweed and biostimulant options based on its demonstrated superior efficacy in previous studies. Its extract has been shown to significantly enhance plant tolerance to abiotic stress by improving amino acid metabolism, antioxidant activity, and nutrient uptake. For example, it outperformed both *Sargassum muticum* and *Ulva lactuca* in promoting growth and metabolic balance in chickpea and fenugreek under stress conditions (Abdel Latef et al., 2017; Battah et al., 2021). Additionally, related species such as *Jania adhaerens* have exhibited strong protective effects against plant pathogens by priming plant defenses and preserving cellular structures (Righini et al., 2022). Thereby, it might be suggested that the exploitation of naturally occurring red algae biomass could offer a substantial relief for plant metal stress, accounting for their pivotal components.

Wheat is among the most widely consumed cereal crops worldwide. Principally, abiotic stresses induce biochemical and physiological changes in plant cells, disturbing their growth, development, and yield (Saad-Allah and Ragab, 2020; Hossain et al., 2021; Duan et al., 2023). Consequently, innovative strategies are required for sustainable wheat cultivation in response to climate change to guarantee food and nutritional security for the growing global population. The present approach was performed to assess the toxic effects of Co stress at morphological, cellular, and subcellular levels in wheat seedlings. The novel potential of *J. rubens* aqueous extract to improve Co stress resilience and mitigate its adverse impacts was further fully explored.

## 2 Materials and methods

### 2.1 Biological material

In April 2023, the dominant red seaweed species *J. rubens* (Linnaeus) Lamouroux was collected from the Rocky Bay of Abu Qir (N 31° 19` E 030° 03`), Alexandria, Egypt (latitudes N 31° 19`N and longitudes 030° 03`E) at a depth of about 1 m. The seaweed was cleaned of debris and stored in seawater in an icebox for transportation to the laboratory. Upon arrival, it was rinsed with tap water and identified using morphological characteristics (Aleem, 1993; Guiry and Guiry, 2019). The sample was air-dried for a day, then oven-dried at 40°C until it reached a constant weight. It was then ground into a fine powder (0.1 mm) and stored in a cool, dark environment for future use.

The grains of *Triticum aestivum* (L.) of cultivar Misr 1 were supplied by the Agricultural Research Center, Giza, Egypt.

### 2.2 Preparation and characterization of aqueous algal extract

The aqueous extracts were prepared by soaking 1 g of algal powder in 100 mL distilled water (w/v) for 4 h at 60 °C according to preliminary experiments. The extract was filtered using Whatman filter paper to obtain a stock solution which was kept at 4°C in the dark for further use.

To estimate the content of phytohormones, *Jania rubens* aqueous extract was first subjected to evaporation within the vacuum chamber of a rotary evaporator. The resulting algal residues were further extracted using methanol (Shindy and Smith, 1975). Quantification of phytohormones including indole-3-acetic acid (IAA), abscisic acid (ABA), gibberellic acid (GA_3_), and cytokinins (zeatin and kinetin derivatives) was performed using gas chromatography (GC, Hewlett Packard, HP 6890 series). Pure analytical standards of each hormone (IAA, ABA, GA_3_, zeatin, and kinetin), obtained from commercial suppliers, were used to construct calibration curves and to identify hormone peaks based on their retention times. The sample concentrations were determined by comparing their peak areas against those of the known standard solutions under the same GC conditions.

For amino acid detection, the dehydrated algal extract residues were defatted with diethyl ether for 24 hours and subsequently air-dried. About 0.2 g of the defatted material was combined with 5 ml of 6 N HCl in a sealed tube and thereafter incubated in an oven at 110°C for 24 hours (Passmore, 1982). The sample was filtered and adjusted to 100 ml with distilled water. The filtrate was dried at 70°C, subsequently re-dissolved in 5 ml of Na-S buffer, filtered, and injected into the amino acid analyzer (SYKAM 5433).

To estimate mineral nutrients in algal extract, the mixed acid–digestion with HNO_3_:H_2_O_2_ mixture (5:3, v/v) was applied according to Allen et al (Allen et al., 1974). The colorimetric test employing Rochelle reagent was performed to quantify the total nitrogen (N) level, whereas the molybdenum blue method (MBM) was applied to ascertain total phosphorus (P) concentration, referencing their standard calibration curves (Allen et al., 1989). The contents of minerals including magnesium (Mg), potassium (K), calcium (Ca), Zinc (Zn), manganese (Mn), iron (Fe), and copper (Cu) were measured with the aid of inductively coupled plasma-optical spectroscopy (Polyscan 61E, Thermo Jarrell-Ash Corp., Franklin, MA, USA).

Moreover, qualitative characterization of crude extract was accomplished using gas chromatography-mass spectrometry (GC-MS). The dehydrated algal residues were subsequently dissolved in 90% ethanol and supplemented with the GC mass spectrometer (Perkin Elmer model: clarus 580/560S). The identification of chemical constituents was recognized according to GC retention time (RT) utilizing a capillary column (Elite-5MS, 30m, 0.25mmID 0.25um df). The obtained mass spectra were compared to a database of standard compounds ranging from 50 to 620 Da.

### 2.3 Experimental design

Wheat (*Triticum aestivum* L.) seeds were surface sterilized using 5% sodium hypochlorite for five minutes, then thoroughly rinsed with distilled water. The sterilized seeds were divided into two priming groups. One group was soaked in distilled water (hydro-priming), and the other in *Jania rubens* extract (*J. rubens* ext.) for 12 hours, followed by thorough rinsing with distilled water to support the physiological foundation of priming as a preconditioning technique to improve plant performance under heavy metal stress.

The experiment was conducted in a controlled greenhouse with a 10-hour photoperiod, temperature regime of 28/16 ± 2°C (day/night), and 60% relative humidity. Primed seeds were sown in plastic pots (20 cm diameter × 15 cm depth) filled with 5 kg of a clay-sandy soil mix (2:1 w/w). Pots were irrigated with tap water until full germination. At 14 days post-sowing, seedlings from both priming treatments were exposed to one of two irrigation regimes at 60% field capacity: distilled water (control) or 150 mM cobalt chloride (CoCl_2_). The CoCl_2_ concentration was pre-determined as sub-lethal through preliminary trials.

Each treatment was replicated across three pots, with each pot serving as an independent biological replicate. For morphological measurements, six randomly selected plants per treatment were analyzed; however, data were averaged per pot to preserve the independence of replicates in statistical analyses.

At 20 days post-sowing, seedlings were harvested, washed with distilled and deionized water and separated into roots and shoots for analysis. Fresh mass (FM) was recorded immediately after harvest, while dry mass (DM) was obtained after drying samples at 60°C until constant weight. Relative water content (RWC) was calculated using the formula: RWC = [(FM − DM)/DM]. Dried samples were finely ground and stored at 4°C for further analysis.

### 2.4 Biochemical analysis

#### 2.4.1 Estimation of photosynthetic parameters, mineral ions, and nitrogenous compounds

To estimate the chlorophylls (Chl) and carotenoid levels, their contents were extracted using 80% acetone in the dark according to Arnon (Arnon, 1949) and Horvath et al (Horvath et al., 1972). The pigment contents were expressed as µg/g FM where the absorbance of the extract was recorded using UV/visible spectrophotometer at 663, 644, and 480 nm for Chl a, Chl b, and carotenoids. Before sample harvesting, the photosynthetic activity of dark-adapted leaves was measured by a digital fluorometer (OS-30 P, Hudson, USA) as photosystem II (PSII) performance (Fv/Fm). Further, the detection of K, Mg, Ca, Fe, and Co ion levels was performed in the digested roots and shoots samples using inductively coupled plasma optics (Allen et al., 1974).

Osmolyte concentrations were determined in plant dry mass using spectrophotometric methods. Proline content was assessed following Bates et al (Bates et al., 1973), where the tissue was homogenized in 3% sulfosalicylic acid and reacted with acid ninhydrin reagent. The resulting chromophore was extracted in toluene and quantified at 520 nm against a proline standard curve. Total amino acids were extracted with 95% ethanol and determined using the ninhydrin-glycerol citrate buffer method described by Lee and Takahashi (1966) with glycine as the standard reference. Glycine betaine was quantified according to Grieve and Grattan (1983) by treating aqueous plant extracts with 2N HCl and cold KI-I2 reagent. The formed periodate crystals were extracted in 1,2-dichloroethane and measured at 365 nm against glycine betaine standards. Results were expressed as mg/g DM for proline and amino acids, and µg/g DM for glycine betaine.

#### 2.4.2 Stress biomarkers determination

For stress markers evaluation, hydrogen peroxide (H_2_O_2_), hydroxyl radical (OH^•^), Malondialdehyde (MDA), and electrolyte conductivity were measured in the fresh leaves. According to (Velikova et al., 2000), H_2_O_2_ content was extracted in 0.1% trichloroacetic acid (TCA) and then mixed with K-phosphate buffer (pH 7.0) and KI_2_ (1M). The mixture absorbance was read at 390 nm and H_2_O_2_ content was measured due to the coefficient of 0.28 µM^–1^ cm^–1^. As for the content of OH•, the leaf tissues were extracted in phosphate buffer (pH 7.4) containing 2-deoxyribose (15 mM) at 37°C as described by (Kaur et al., 2012). The extract (0.7 ml) was boiled for 30 min with (3:1) mixture of 0.5% (w/v) thiobarbituric acid (TBA) and glacial acetic acid. The mixture was cooled, and the absorbance was recorded at 532 nm and 600 nm. With the aid of an extinction coefficient of 155 mM^-1^ cm^-1^, the ^•^OH was expressed as µmol/g FM. To determine the lipid peroxidation product (MDA), the samples were mixed in 5% (w/v) TCA (Heath and Packer, 1968). The yielded extract was incubated with 0.67% (w/v) TBA at 100°C for 20 min then instantly cooled. Absorbance was detected at 532 and 600 nm and the extinction coefficient (155 mM^-1^ cm^-1^) was employed to calculate MDA (µmol/g FM). The electrical conductivity (EC) (µmohs/cm) was measured in a solution of deionized water mixed with a definite weight of fresh leaf tissues after 1 h as EC1 and after 24 h as EC2. To express the electrolyte/membrane leakage (EL) as a percentage the formula EC1/EC2×100 was applied (Sairam et al., 2005).

#### 2.4.3 Estimation of antioxidant enzymes

Antioxidant enzyme activities were determined in fresh leaf samples. The enzyme extraction was performed by homogenizing 0.5 g tissue in pre-chilled K-phosphate buffer (pH 7), followed by centrifugation at 10,000 rpm for 20 min. Guaiacol peroxidase (GPOX, EC 1.11.1.7) activity was assayed according to Kato and Shimizu (1987) by monitoring guaiacol oxidation by H_2_O_2_ at 470 nm in K-phosphate buffer (pH 5.8), using an extinction coefficient of 26.6 mM^–1^ cm^–1^. Ascorbate peroxidase (APOX, EC 1.11.1.11) activity was determined following Nakano and Asada (1981) by measuring H_2_O_2_-dependent ascorbate oxidation at 290 nm (ϵ = 2.8 mM^–1^ cm^–1^) in a reaction mixture containing ascorbic acid, H_2_O_2_, and EDTA in K-phosphate buffer (pH 7). Glutathione reductase (GR, EC 1.6.4.2) activity was assessed by monitoring NADPH oxidation at 340 nm (ϵ = 6.2 mM^–1^ cm^–1^) in a reaction mixture containing K-phosphate buffer (pH 7.5), EDTA, NADPH, and GSSG (Halliwell and Foyer, 1978).

Additional antioxidant enzyme activities were measured spectrophotometrically. Polyphenol oxidase (PPO, EC1.10.3.1) activity was determined according to Kumar and Khan (1982) by monitoring tannic acid oxidation at 420 nm (ϵ = 26.40 mM^–1^ cm^–1^) in K-phosphate buffer (pH 7), using an extinction coefficient of 40 mM^–1^ cm^–1^) in K-phosphate buffer (pH 6.0) after 5 min incubation at 25°C and termination with 2N H_2_SO_4_. Superoxide dismutase (SOD, EC 1.15.1.1) activity was assessed following (Beyer Jr. and Fridovich, 1987) by measuring the photochemical reduction of nitroblue tetrazolium (NBT) at 560 nm (ϵ = 21.1 mM^–1^ cm^–1^) in a reaction mixture containing L-methionine, NBT, Triton X-100, and riboflavin in K-phosphate buffer (pH 7.8) under fluorescent illumination for 15 min. Catalase (CAT, EC1.11.1.6) activity was quantified by monitoring H_2_O_2_ decomposition at 240 nm (Kato and Shimizu, 1987) in K-phosphate buffer (pH 7), using an extinction coefficient of 40 mM^–1^ cm^–1^.

#### 2.4.4 Estimation of non-enzymatic antioxidants

Non-enzymatic antioxidants were quantified in fresh leaf tissues. Ascorbic acid (ASA) was extracted in 5% sulfosalicylic acid following Oser (Oser, 1979), and determined spectrophotometrically at 660 nm after reaction with Na-molybdate (2%), H_2_SO_4_ (0.15N), and Na_2_HPO_4_ (1.5 mM) at 60°C for 40 min. Reduced glutathione (GSH) was extracted from fresh leaves (0.1 g) in 5% sulfosalicylic acid and quantified according to Andersen (Andersen, 1985) by measuring the reaction with 5,5′-dithiobis (2-nitrobenzoic acid) in K-phosphate buffer (pH 7) containing EDTA at 412 nm. Both ASA and GSH contents were calculated using their respective standard curves and expressed as mg/g FM and µg/g FM, respectively.

Furthermore, the dry shoots were extracted in absolute ethanol to estimate total flavonoids (FA) and phenolics (Ph) levels. According to Chang et al (Chang et al., 2002), flavonoids were detected by mixing 0.5 ml plant extract with a mixture of 10% AlCl_3_, 1M K-acetate, and 95% ethanol. After 30 min, the absorbance was detected at 417 nm to express flavonoids against a standard of quercetin. According to Jindal et al (Jindal and Singh, 1975), phenolics were determined by adding 0.1 plant extract to a mixture of 0.1 ml Folin-Ciocalteu’s reagent and 1 ml 20% Na_2_CO_3_. After 1 h, the absorbance was measured at 650 nm against standard phenol to quantify phenolics (mg/g DM).

#### 2.4.5 Ultra-structure analysis

For transmission electron microscope (TEM) analysis, the seedling leaves and roots were washed carefully with distilled water to eliminate any residues. Definite leaf sectors were initially fixed in 4% aqueous glutaraldehyde (v/v) and then immersed in 1% potassium permanganate solution for 5 min at room temperature. The specimens were then washed with distilled water three times and further dehydrated by ethanol series. The dehydrated samples were embedded in an epoxy resin and prepared for sectioning. The Ultrathin sections (50–80 nm thick) were mounted on copper grids and stained with uranyl acetate and Reynold’s lead citrate (Reynolds, 1963). The stained ultrathin sectors were examined with a JEOL 1010 Transmission Electron Microscope (JEOL, Peabody, MA).

### 2.5 Statistical analysis

Data analysis was performed using GraphPad Prism^®^ (version 8.3.0) and SPSS (v.23) software. One-way ANOVA followed by Tukey’s HSD test (P ≤ 0.05) was used to assess differences between treatments. Pearson’s correlation analysis was conducted to evaluate relationships between growth parameters, mineral content, antioxidant systems, and phytochemical compounds. Results are presented as means ± standard deviation (SD).

## 3 Results

### 3.1 Phytochemical characterization of *J. rubens* extract

The phytochemical characterization of *J. rubens* extract revealed its diverse compositional profile (**Table 1**). The amino acid analysis showed that aspartic acid was the most abundant amino acid (50.1 mg/g DM), followed by lysine (25.5 mg/g DM) and tyrosine (22.4 mg/g DM), while methionine and cysteine were present in the lowest concentrations. Regarding mineral composition, potassium was the predominant element (325.9 mg/g DM), followed by significant levels of total nitrogen (83.5 mg/g DM), magnesium (28.4 mg/g DM), calcium (23.5 mg/g DM), and phosphorus (8.1 mg/g DM). The extract also contained detectable amounts of micronutrients including iron, zinc, copper, and manganese. Additionally, several phytohormones were identified, with gibberellic acid (0.166 mg/g) and kinetin (0.144 mg/g) being the most abundant, while abscisic acid was present in trace amounts.

**Table 1:** Phytochemical characterization of *J. rubens*.

Amino acids			Phytohormones (mg/g	
Essential	Concentration (µg/g)	%		
Arginine	15.5 ± 1.3	4.7	Indole acetic acid	0.035 ± 0.003
Histidine	11.3 ± 0.35	3.4	Gibberellic acid	0.166 ± 0.008
Isoleucine	11.8 ± 0.36	3.6	Zeatin	0.026 ± 0.003
Lysine	25.5 ± 1.1	7.7	Kinetin	0.144 ± 0.004
Phenylalanine	11.8 ± 0.99	3.6	Abscisic acid	0.005 ± 0.001
Threonine	16.4 ± 3.1	5.0	Mineral nutrients ions (mg/g)	
Valine	13.3 ± 2.0	4.0		
Methionine	1.4 ± 0.1	0.4		
Leucine	20.7 ± 2.3	6.3		
Non-essential			Nitrogen	83.5 ± 4.61
Aspartic acid	50.1 ± 4.8	15.2	Phosphorous	8.1 ± 0.36
Serine	17.5 ± 3.9	5.3	Potassium	325.9 ± 10.0
Glutamic acid	40.7 ± 4.5	6.2	Magnesium	28.4 ± 3.85
Proline	12.7 ± 1.5	3.8	Calcium	23.5 ± 2.96
Glycine	20.6 ± 0.7	6.2	Zinc	0.123 ± 0.012
Alanine	19.2 ± 1.9	5.8	Iron	0.35 ± 0.03
Tyrosine	22.4 ± 2.5	6.8	Copper	0.082 ± 0.00
Cysteine	4.8 ± 0.3	1.4	Manganese	0.051 ± 0.00

### 3.2 Qualitative GC–MS analysis of *J. rubens* extract

The major phytochemical constituents, identified via GC analysis, are listed in **Table 2** according to the obtained chromatogram (**Supplementary Figure S1**). The GC-MS analysis of *J. rubens* aqueous extract revealed nine major compounds, with fatty acids being the predominant class of constituents (**Table 2**). Among the fatty acids, cis-vaccenic acid was the most abundant component, representing 33.76% of the total composition, followed by hexadecanoic acid at 14.47% and octadecanoic acid at.10.01%. The extract also contained ester derivatives, including 2-pentanone,4-hydroxy-4-methyl-ester (22.1%), 10-undecenoic acid butyl ester (6.61%), and hexadecanoic acid,2-hydroxy-1-(hydroxymethyl)ethyl ester (2.42%). Additionally, the analysis identified an aldehyde compound (9,17-octadecadienal, 3.70%) and a cyclic compound (undecanoic acid hydroxy-lactone, 5.26%).

**Table 2:** GC-MS analysis of the active constituents in aqueous extract of *J. rubens*.

No	Retention time	Compounds	Molecular formula	Content (%)
1	27.423	cis-Vaccenic acid	C_18_H_34_O_2_	33.67%
2	4.009	2-Pentanone, 4-hydroxy-4-methyl-ester	C_6_H_12_O_2_	22.10%
3	25.032	Hexadecanoic acid	C_16_H_32_O_2_	14.47%
4	27.708	Octadecanoic acid	C_18_H_36_O_2_	10.01%
5	32.145	10-Undecenoic acid, butyl ester	*C_15_H_28_O_2_*	6.61%
6	31.285	Undecanoic acid, hydroxy-, lactone	C_11_H_18_O_2_	5.26%
7	30.680	9,17-Octadecadienal	C_18_H_32_O	3.70%
8	28.609	Hexadecanoic acid, 2-hydroxy-1-(hydroxymethyl)ethyl ester	C_19_H_38_O	2.42%
9	29.134	Octadecanoic acid	C_18_H_36_O_2_	1.77%

### 3.3 Growth characteristics

**Table 3** illustrates the detrimental impact of cobalt (Co) stress on wheat seedling growth and the mitigating effect of *J. rubens* extract. Exposure to 150 mM CoCl_2_ significantly reduced key shoot parameters, including shoot length (20.6 cm), dry weight (0.048 g), relative water content (85.4%), and leaf area (4.1 cm²), compared to control plants (28.1 cm, 0.063 g, 89.9%, and 6.0 cm², respectively). Root traits were also impaired, with root length and dry weight decreasing from 19.8 cm and 0.033 g in control plants to 10.9 cm and 0.019 g under Co stress.

**Table 3:** Growth parameters of wheat (*Triticum aestivum* L.) seedlings subjected to cobalt stress (150 mM CoCl_2_) and pretreated with *J. rubens* extract (1% w/v, 12 h soaking).

Treatments	Length (cm/organ)		Biomass (g Dw/organ)		Relative water content (%)		Leaf area (cm^2^/leaf)
	Root	Shoot	Root	Shoot	Root	Shoot	
Control	19.8 ± 0.4^b^	28.1 ± 2.1^a^	0.033 ± 0.002^a^	0.063 ± 0.003^b^	87.5 ± 1.0^b^	89.9 ± 0.4^a^	6.0 ± 0.3^a^
Co stress	10.9 ± 0.5^d^	20.6 ± 0.8^c^	0.019 ± 0.003^b^	0.048 ± 0.001^d^	91.8 ± 1.5^a^	85.4 ± 1.2^b^	4.1 ± 0.3^b^
*J. rubens* ext.	23.0 ± 2.3^a^	28.9 ± 1.0^a^	0.034 ± 0.002^a^	0.076 ± 0.004^a^	90.2 ± 2.1^ab^	89.1 ± 0.7^a^	6.1 ± 0.5^a^
*J. rubens* ext.+ Co	17.0 ± 1.5^c^	25.3 ± 1.8^b^	0.031 ± 0.003^a^	0.058 ± 0.002^c^	92.4 ± 0.8^a^	88.9 ± 1.1^a^	5.4 ± 0.9^a^

Values are expressed as mean ± SE (n = 3 biological replicates). Different superscript letters within a row indicate significant differences according to Tukey’s HSD test (p < 0.05).

Priming with *J. rubens* extract effectively alleviated these negative effects. Under Co stress, *J. rubens* pretreatment improved shoot length to 25.3 cm and restored shoot dry weight to 0.058 g values approaching those of the untreated control. Similarly, root length and biomass increased to 17.0 cm and 0.031 g, while leaf area expanded to 5.4 cm². Notably, relative water content in both roots and shoots remained high (92.4% and 88.9%, respectively).

**Figure 1:**
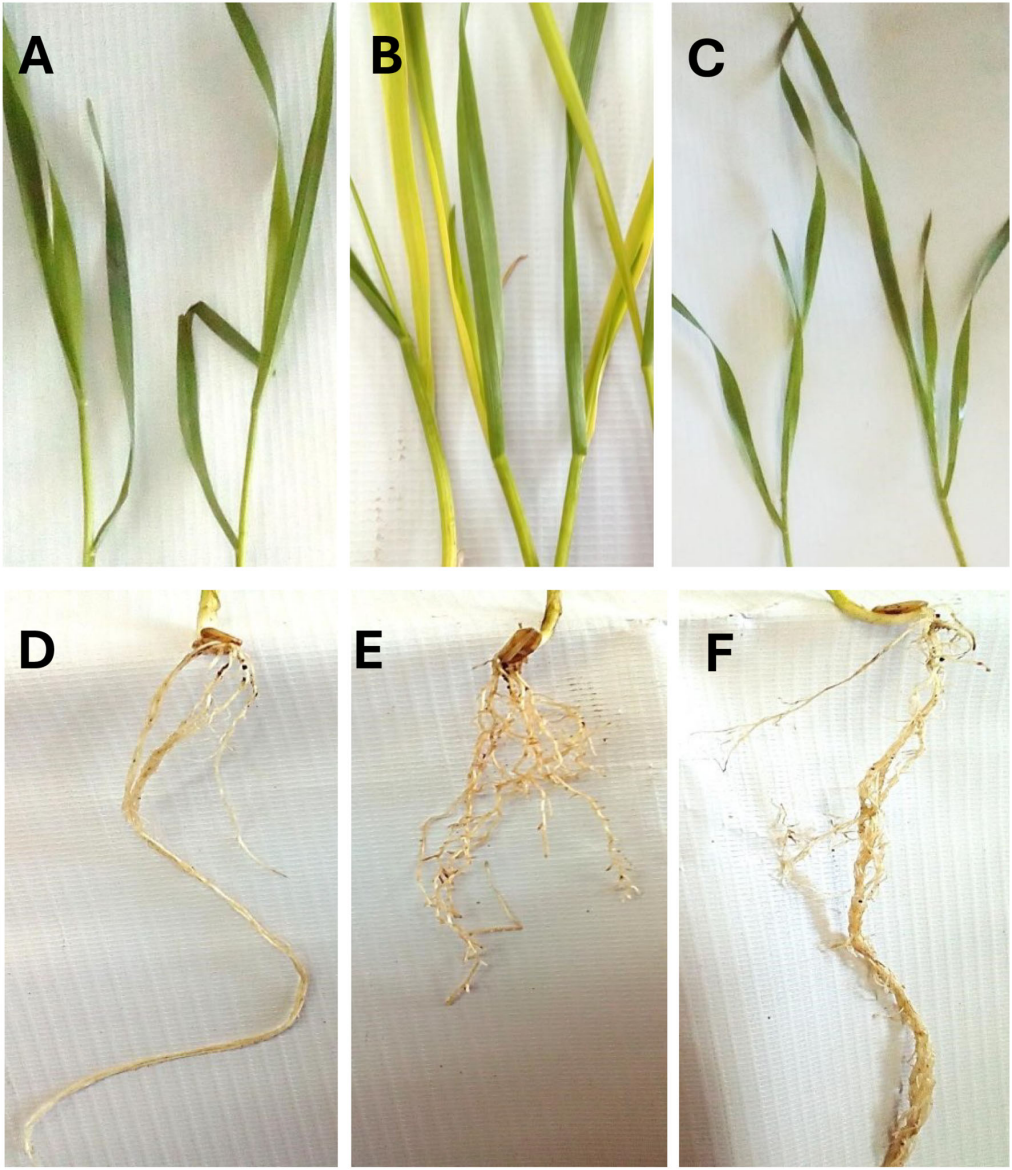
Morphological symptoms of wheat seedlings exposed to cobalt stress (150 mM CoCl_2_) and presoaked in *J. rubens* extract (1% w/v, 12 h soaking), **(A, D)** control, **(B, E)** Co stress, and **(C, F)** combined treatment of Co stress and *J. rubens* extract.

Moreover, the exposure to Co stress resulted in distinct morphological symptoms (**Figure 1**) including severe leaf chlorosis and stunted roots with thickened abnormal branching patterns. These morphological abnormalities were considerably limited by priming in *J. rubens* extract, clarifying its alleviatory impact (**Figures 1C, F**).

### 3.4 Photosynthetic pigments and photosynthetic performance (Fv/Fm)

The effect of *J. rubens* extract and cobalt stress on the contents of Chl a, b, and carotenoids, as well as photosynthetic activity in *T. vulgare* seedlings is illustrated in **Table 4**. Cobalt stress caused a considerable reduction in photosynthetic pigment levels: Chl a by 32.6%, Chl b by 40%, and carotenoids by 30.15%, respectively, relative to non-treated plants.

**Table 4:** Photosynthetic characteristics (chlorophyll a, b, total chlorophyll, and carotenoids) in leaves of wheat seedlings exposed to 150 mM CoCl_2_ and primed *with J. rubens* extract (1% w/v, 12 h).

Treatments	Chl a (µg/g FM)	Chl b (µg/g FM)	Carotenoids (µg/g FM)	Photosynthetic activity
Control	366.1 ± 18^b^	123.6 ± 17^b^	148.9 ± 11^a^	0.785 ± 0.009^a^
Co stress	246.8 ± 28^c^	74.2 ± 12^c^	104.0 ± 13^b^	0.714 ± 0.010^c^
*J. rubens* ext.	440.7 ± 40^a^	153.3 ± 14^a^	163.2 ± 14^a^	0.790 ± 0.006^a^
*J. rubens* ext.+ Co	450.2 ± 35^a^	157.2 ± 16^a^	159.5 ± 8.6^a^	0.764 ± 0.003^b^

Values represent means ± SE (n = 3 biological replicates). Different superscript letters denote significant differences according to Tukey’s HSD test (p < 0.05).

However, *J. rubens* extract application under cobalt stress resulted in a significant increase in Chl a (82.4%), Chl b (111.86%), and carotenoids (53.37%) compared to cobalt stress treatment alone. Moreover, cobalt stress negatively affected the photosynthetic machinery and decreased PSII activity by 9% compared to the non-stressed plants. Nevertheless, pre-soaking with *J. rubens* extract significantly influenced the chlorophyll fluorescence parameter by improving PSII efficiency by 7% under cobalt stress conditions.

Additionally, the application of *J. rubens* extract to unstressed plants showed a substantial increase in photosynthetic attributes compared to the control, demonstrating its stimulating activity. Notably, the highest values of Chl a (450.2 ± 35 µg/g FM), Chl b (157.2 ± 16 µg/g FM), and carotenoids (159.5 ± 8.6 µg/g FM) were observed in plants treated with *J. rubens* extract under cobalt stress, which were statistically similar to those treated with *J. rubens* extract alone.

### 3.5 Mineral analysis

**Figure 2:**
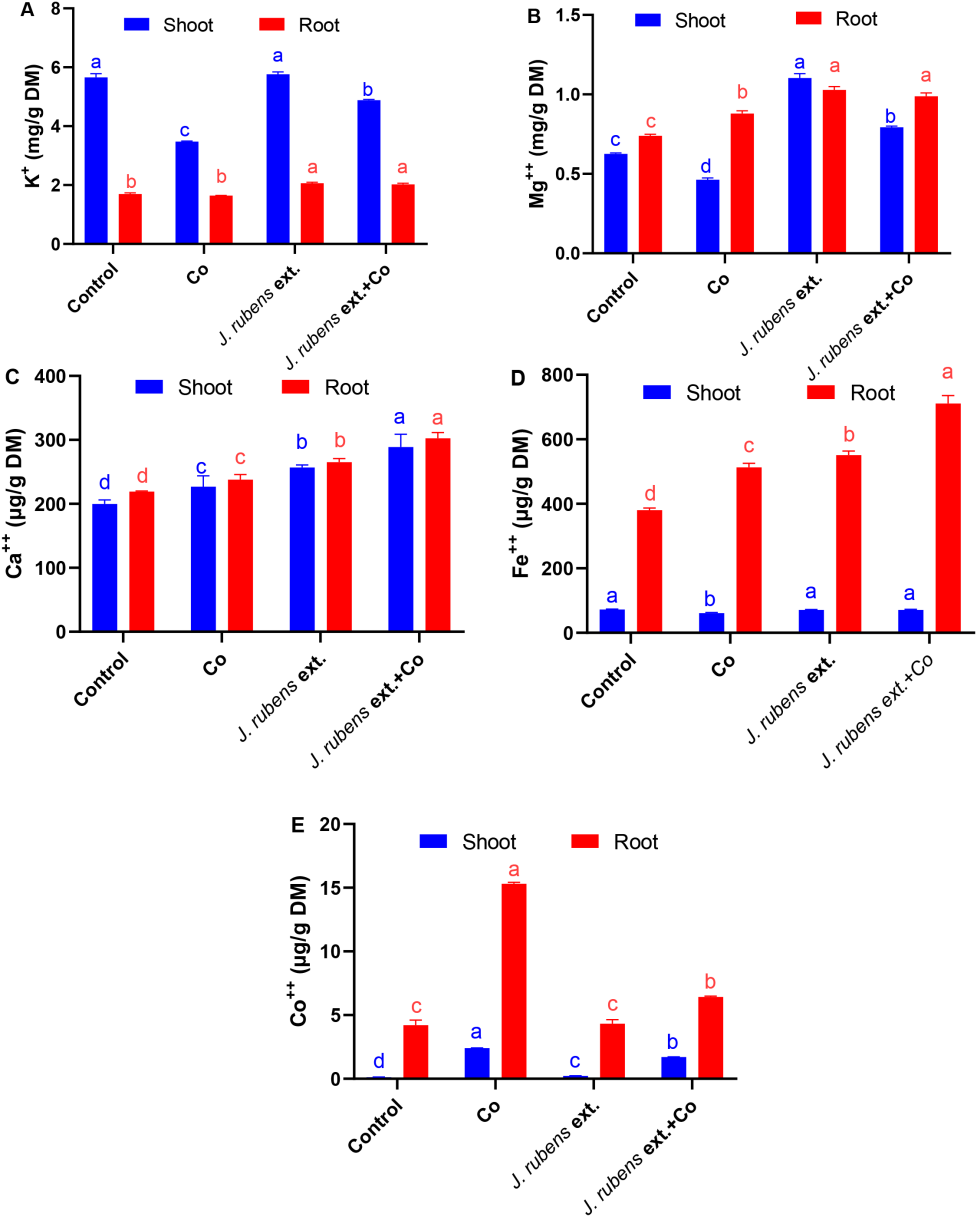
Mineral content in roots and shoots of wheat seedlings subjected to cobalt stress (150 mM CoCl_2_) with or without *J. rubens* priming (1% w/v, 12 h). Potassium **(A)**, magnesium **(B)**, calcium **(C)**, iron **(D)**, and cobalt **(E)**. Values represent means ± SE (n = 3). Statistically significant differences are marked by different letters (p < 0.05, HSD test).

Mineral status in wheat seedlings is illustrated in **Figure 2**. Cobalt stress significantly reduced the uptake of K, Mg, and Fe in shoot tissues by 38.5%, 27%, and 15.6%, respectively; however, their levels in stressed roots were either elevated or non-significantly changed when compared to control plants. Notably, a significant increase in Ca contents in both roots and shoots was documented following Co stress application, by 8.68% and 13.5%, respectively, relative to the control (**Figure 2**).

Pre-soaking with *J. rubens* extract, under both control and stressed conditions, exhibited a stimulatory effect on the mineral status and significantly enhanced K, Mg, and Fe levels in both root and shoot tissues compared to Co stress treatment alone. Interestingly, Ca content also increased in both organs following *J. rubens* extract application, surpassing levels observed in both the Co-only and control treatments.

Regarding Co content, irrigation with CoCl_2_ pronouncedly increased its accumulation in wheat seedlings, with significantly higher concentrations in roots (15.3 ± 0.1 µg/g DM) compared to shoots (2.41 ± 0.01 µg/g DM). The alleviatory effect of *J. rubens* extract was evident in its ability to reduce Co concentration in stressed roots and shoots by 58.17% and 29.05%, respectively, compared to Co stress treatment alone.

These data demonstrate that *J. rubens* extract application resulted in the highest values for most essential minerals (K, Mg, Ca, and Fe) in both roots and shoots, while simultaneously reducing toxic Co accumulation in plant tissues under stress conditions. The most substantial improvement was observed in root Fe content, which reached 711 ± 24 µg/g DM in the combined *J. rubens* extract and Co treatment, representing an 87% increase compared to control and a 39% increase compared to Co stress alone.

### 3.6 Oxidative stress biomarkers

**Figure 3:**
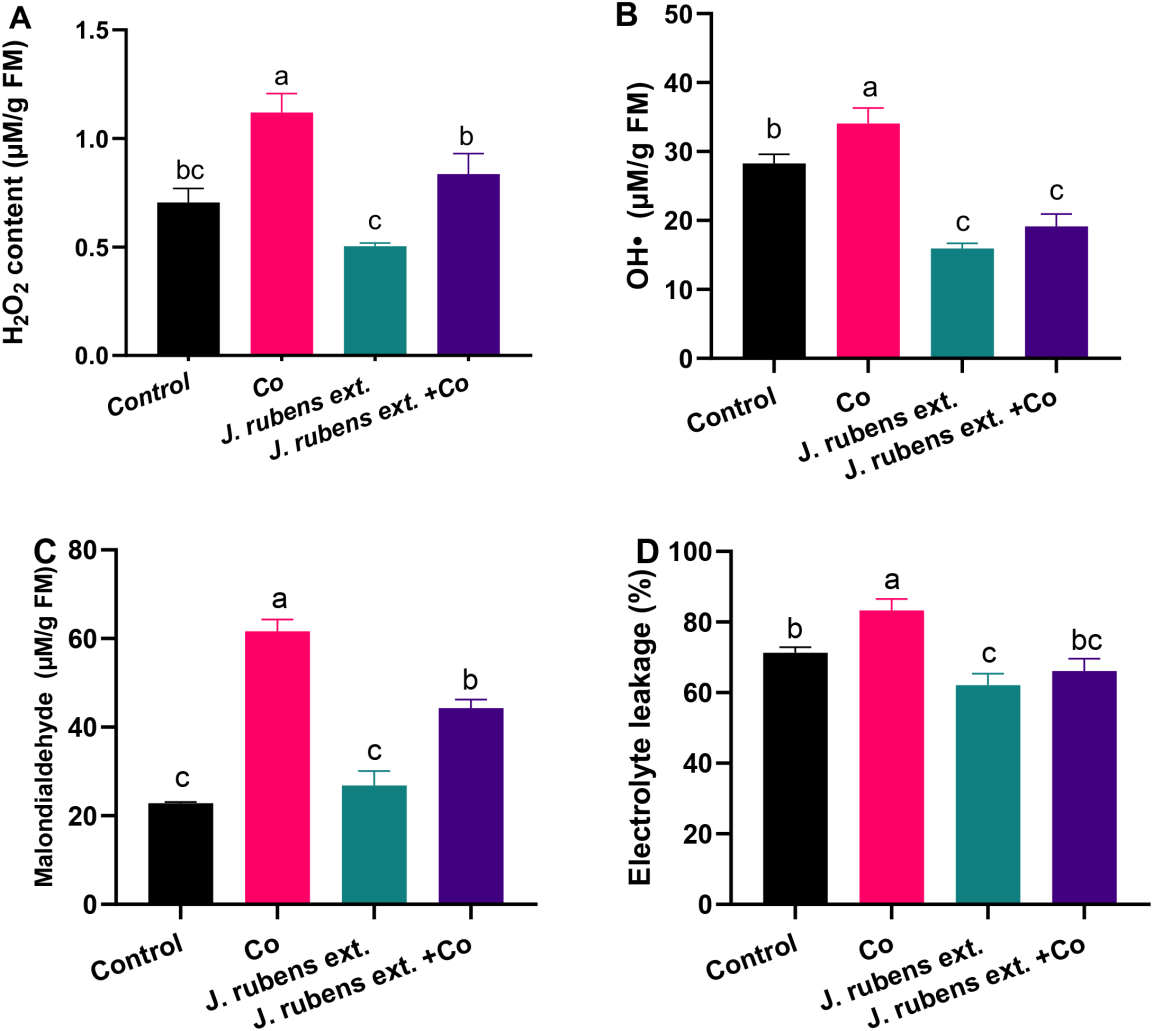
Oxidative stress markers in leaves of wheat seedlings primed with *J. rubens* extract (1% w/v, 12 h) and exposed to cobalt stress (150 mM CoCl_2_). **(A)** H_2_O_2_ content, **(B)** Hydroxyl radicals, **(C)** Malondialdehyde, and **(D)** Electrolyte leakage. Values represent means ± SE (n = 3). Statistically significant differences are marked by different letters (p < 0.05, HSD test).

Cobalt stress significantly elevated oxidative stress biomarkers in wheat seedlings compared to the control, clearly demonstrating the toxicity of cobalt ions (**Figure 3**). Specifically, the levels of hydrogen peroxide (H_2_O_2_), hydroxyl radicals (OH•), malondialdehyde (MDA), and electrolyte leakage (EL) exhibited substantial increases of 67.1%, 20.5%, 170.1%, and 16.9%, respectively, relative to the control conditions.

The application of *J. rubens* extract as a presoaking treatment demonstrated remarkable effectiveness in mitigating cobalt-induced oxidative damage. This protective effect was evidenced by the significant reduction in H_2_O_2_ content, OH• production, lipid peroxidation (measured as MDA), and membrane integrity disruption (measured as electrolyte leakage) in cobalt-stressed seedlings pretreated with the extract.

Notably, the *J. rubens* extract treatment reduced H_2_O_2_ levels in cobalt-stressed plants by approximately 25% compared to cobalt stress alone (**Figure 3A**). Even more striking was the effect on hydroxyl radical levels, where the extract reduced OH• concentrations in stressed plants by approximately 42% compared to cobalt stress treatment, bringing the levels even below those of the control group (**Figure 3B**).

Lipid peroxidation, a key indicator of oxidative damage to cell membranes measured as MDA content, was significantly decreased by approximately 30% in the *J. rubens* extract + Co treatment compared to cobalt stress alone (**Figure 3C**). Similarly, electrolyte leakage, which reflects membrane integrity, was reduced to levels comparable to the control when *J. rubens* extract was applied to cobalt-stressed seedlings (**Figure 3D**).

### 3.7 Antioxidant enzyme activities

**Figure 4:**
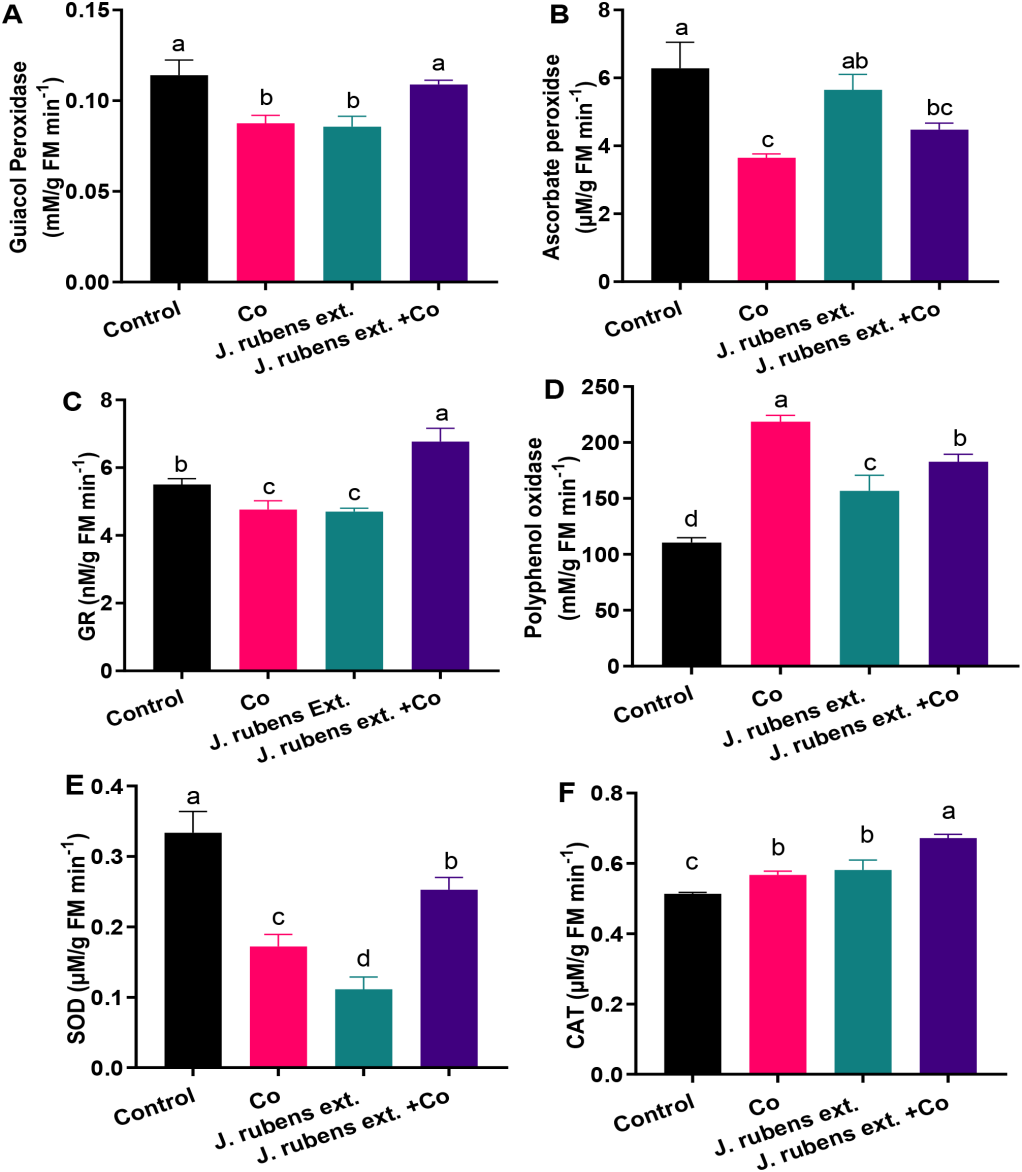
Antioxidant enzyme activities in leaves of wheat seedlings treated with *J. rubens* extract (1% w/v, 12 h) and exposed to 150 mM CoCl_2_. **(A)** Guaiacol peroxidase, **(B)** Ascorbate peroxidase, **(C)** Glutathione reductase, **(D)** Polyphenol oxidase, **(E)** Superoxide dismutase, and **(F)** Catalase. Data are meant ± SE (n = 3). Statistically significant differences are marked by different letters (p < 0.05, HSD test).

Exposure to cobalt stress led to significant reductions in the activities of several key antioxidant enzymes compared to control conditions (**Figure 4**). Specifically, superoxide dismutase (SOD) and ascorbate peroxidase (APOX) activities decreased dramatically by 48.5% and 42%, respectively (**Figures 4E, B**). Similarly, cobalt stress reduced the activities of guaiacol peroxidase (GPOX) and glutathione reductase (GR) by 22.81% and 13.61%, respectively, compared to control plants (**Figures 4A, C**).

Pre-soaking wheat seedlings with *J. rubens* extract effectively mitigated cobalt-induced inhibition of antioxidant enzyme activities. This protective effect was particularly evident for GR, where *J. rubens* extract treatment not only reversed the cobalt-induced reduction but increased activity beyond control levels. The stimulatory effect of *J. rubens* extract + Co on GR activity was approximately 50% higher than control levels and approximately 70% higher than cobalt stress alone (**Figure 4C**).

In contrast to the inhibitory pattern observed with SOD, APOX, GPOX, and GR, cobalt stress substantially increased polyphenol oxidase (PPO) activity by 98% and catalase (CAT) activity by 10.31% compared to control plants (**Figures 4D, F**). Treatment with *J. rubens* extract further enhanced the activities of these enzymes to levels significantly exceeding those of both cobalt-stressed and control plants.

Notably, the application of *J. rubens* extract alone (without cobalt stress) resulted in the lowest SOD activity among all treatments (**Figure 4E**), suggesting possible compensatory mechanisms where other antioxidant systems might be upregulated. However, when combined with cobalt stress, *J. rubens* extract significantly restored SOD activity to intermediate levels between control and cobalt-stressed plants.

### 3.8 Antioxidant compounds contents

**Figure 5:**
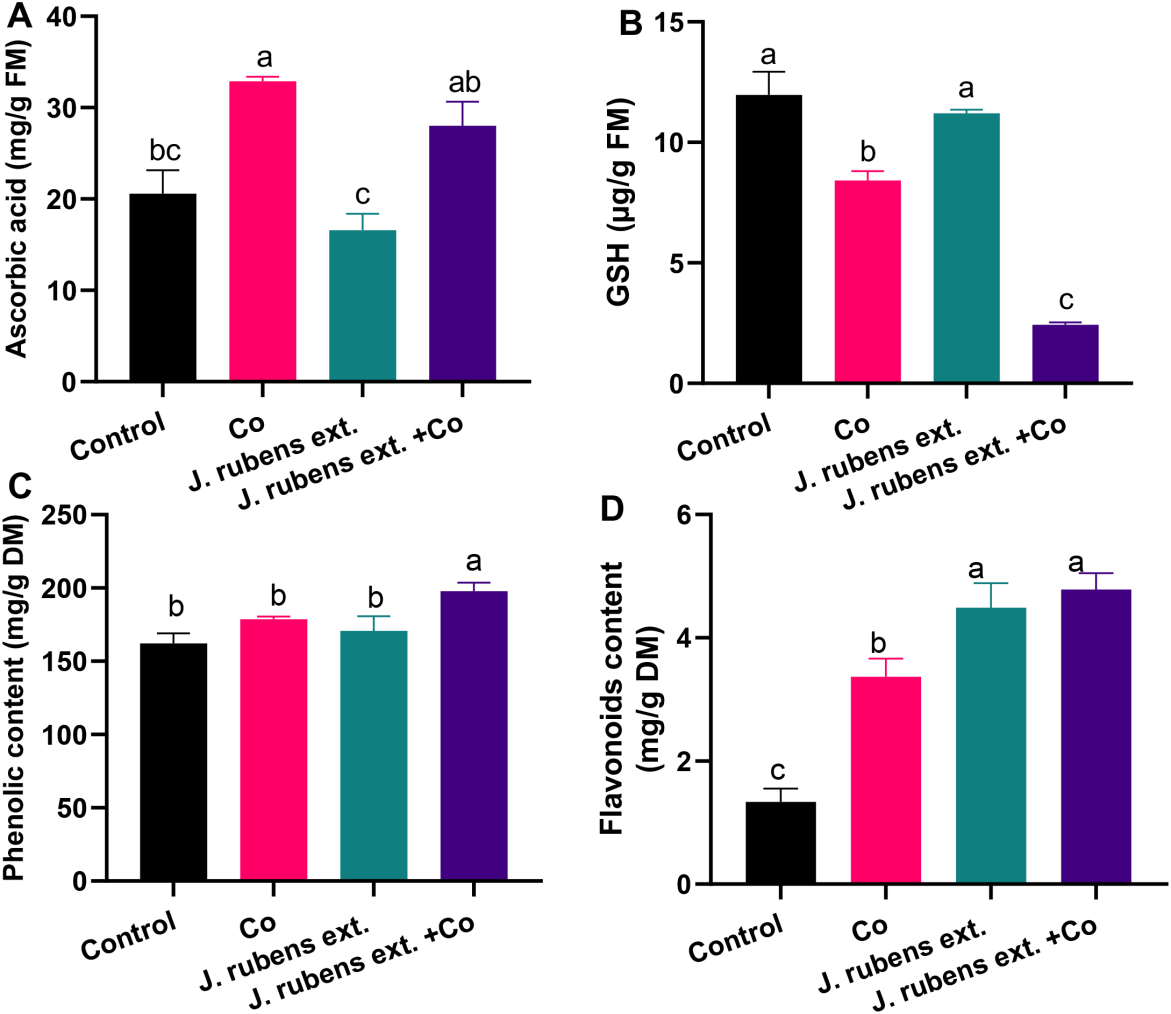
Non-enzymatic antioxidant compounds in leaves of wheat seedlings pretreated with *J. rubens* extract (1% w/v, 12 h) and subjected to 150 mM CoCl_2_. **(A)** Ascorbic acid, **(B)** Glutathione, **(C)** Phenolics, and **(D)** Flavonoids. Data represent means ± SE (n = 3). Statistically significant differences are marked by different letters (p < 0.05, HSD test).

As shown in **Figure 5**, treating wheat seedlings with CoCl_2_ significantly altered the levels of various non-enzymatic antioxidant compounds. Cobalt stress decreased glutathione (GSH) levels by 30% compared to control conditions (**Figure 5B**), while simultaneously enhancing ascorbic acid (ASA) and total flavonoids by 59.7% and 151.5%, respectively (**Figures 4A, D**). The phenolic content under cobalt stress also showed a moderate increase of approximately 12% compared to control plants (**Figure 5C**).

Pre-soaking wheat seedlings with *J. rubens* extract prior to cobalt exposure resulted in differential modulation of these antioxidant compounds. The treatment significantly increased total phenolics and flavonoid levels by 10.74% and 42.14%, respectively, beyond the levels observed under cobalt stress alone (**Figures 5C, D**). Additionally, the *J. rubens* extract + Co treatment enhanced ASA content by 35.9% compared to the untreated control, though this level was somewhat lower than in seedlings exposed to cobalt stress alone (**Figure 5A**).

Interestingly, the application of *J. rubens* extract, both alone and in combination with cobalt stress, resulted in a further reduction of GSH levels compared to both cobalt stress and control treatments (**Figure 5B**).

The most pronounced effect of *J. rubens* extract was observed in flavonoid accumulation, where levels in both extract-treated groups (*J. rubens* extract alone and *J. rubens* extract + Co) were approximately three times higher than control levels (**Figure 5D**).

### 3.9 Nitrogenous compounds content

**Figure 6:**
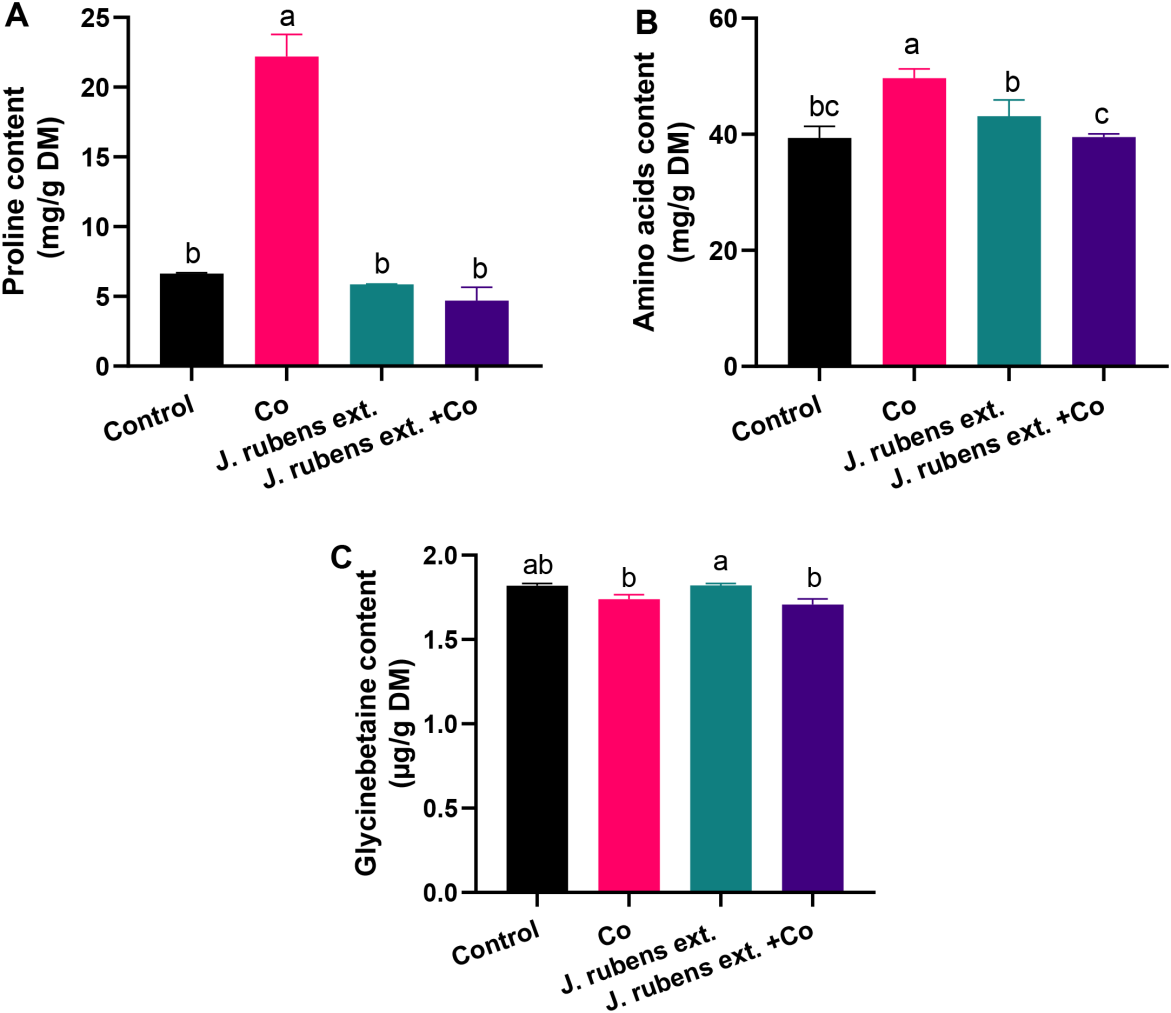
Nitrogenous osmolytes in leaves of wheat seedlings pretreated with *J. rubens* extract (1% w/v, 12 h) under cobalt stress (150 mM CoCl_2_). Measurements were taken from the leaves. Values are means ± SE (n = 3). **(A)** Proline, Total free amino acids **(B)**, and **(C)** Glycine betaine. Letters indicate significant differences (p < 0.05, HSD).

The data presented in **Figure 6** demonstrates distinct changes in nitrogenous compound accumulation in wheat seedlings under cobalt stress and *J. rubens* extract treatments. Cobalt stress irrigation resulted in dramatic increases in both proline and total free amino acids compared to control conditions. Specifically, proline content increased by 234.7% (**Figure 6A**) and amino acids by 26.14% (**Figure 6B**) relative to untreated control seedlings.

The application of *J. rubens* extract had a notable normalizing effect on these stress-induced changes. When applied to cobalt-stressed seedlings, *J. rubens* extract significantly reduced proline and amino acid contents to levels comparable to the control. This reduction was particularly pronounced for proline, where *J. rubens* extract treatment completely counteracted the cobalt-induced accumulation, bringing levels down by approximately 76% compared to cobalt stress alone (**Figure 6A**).

Regarding glycine betaine (GB), cobalt stress caused a slight decrease in its content compared to control conditions, though this reduction was not statistically significant (**Figure 6C**). Interestingly, seedlings treated with *J. rubens* extract alone showed the highest GB content among all treatments, though this was only significantly different from the cobalt stress and combined treatment groups, not from the control.

### 3.10 Correlation analysis

**Figure 7:**
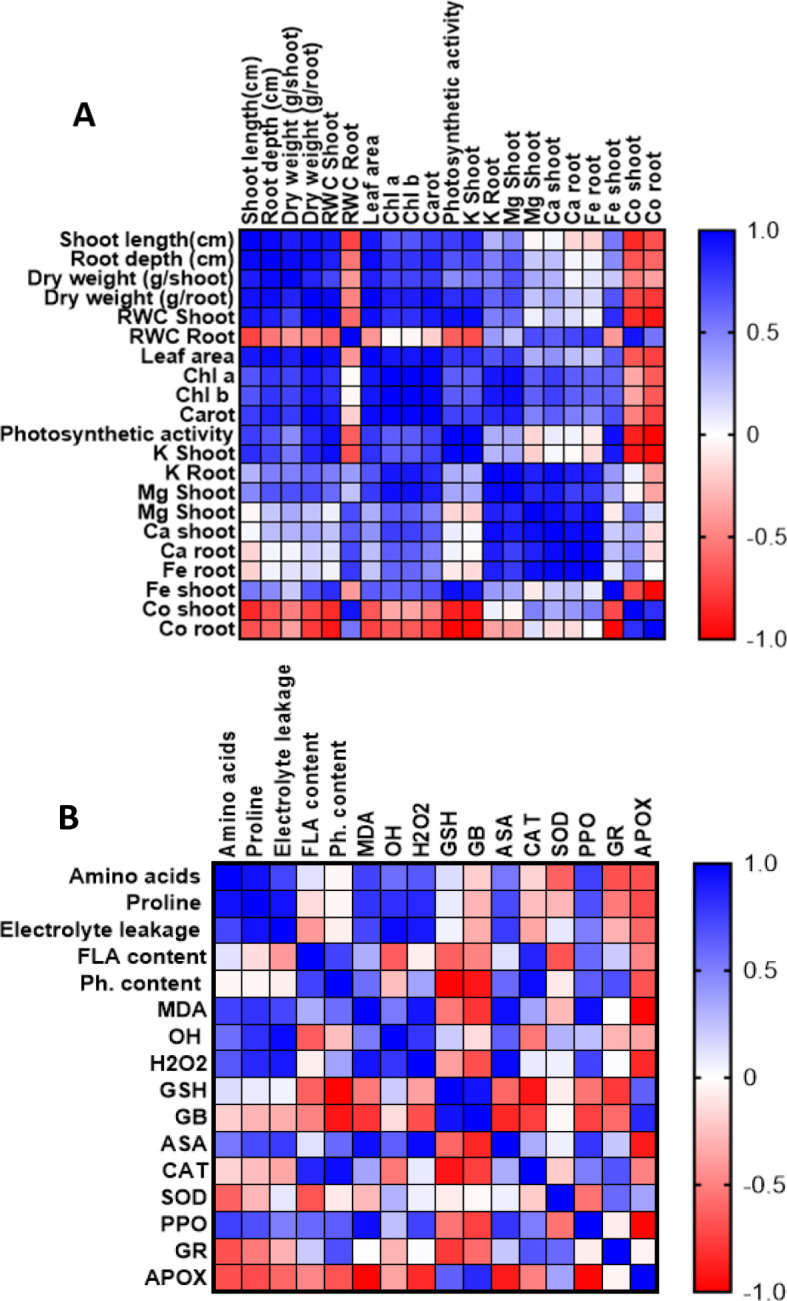
Correlation heatmap illustrating the relationships between morphological, physiological, and nutritional parameters **(A)**, and between stress markers and defense mechanisms **(B)** in wheat leaves. The analysis is based on three biological replicates per treatment. Treatments included cobalt chloride (150 mM) stress, *J. rubens* extract priming (1% w/v), and their combination. Color gradients indicate Pearson correlation coefficients ranging from +1.0 (strong positive; blue) to –1.0 (strong negative; red).

The correlation analyses revealed complex interactions between growth parameters, physiological responses, and stress defense mechanisms under cobalt stress conditions with Jania extract treatment (**Figures 7A, B**).

As shown in **Figure 7A**, strong positive correlations (r ≈ 1.0) were observed among morphological parameters, including shoot length, root depth, and dry weight, indicating synchronized growth responses. Leaf area exhibited significant positive correlations with both shoot and root growth parameters, while relative water content in both organs maintained positive correlations with growth metrics, suggesting preserved water relations. The photosynthetic apparatus showed strong positive correlations between chlorophyll (a, b) content and photosynthetic activity, with carotenoids displaying similar positive correlations. Notably, all photosynthetic parameters positively correlated with growth metrics, indicating maintained photosynthetic efficiency under stress conditions. Regarding mineral nutrition, K content in both shoots and roots demonstrated positive correlations with growth and photosynthetic parameters, while Mg and Ca showed moderate positive correlations with physiological parameters. Fe content exhibited variable correlations, suggesting complex interactions under cobalt stress. Importantly, cobalt accumulation showed strong negative correlations with most measured parameters.

The antioxidant-metabolite correlation analysis revealed significant interactions in stress response mechanisms (**Figure 6B**). Amino acids and proline demonstrated strong positive correlations with each other and with antioxidant markers (GSH, ASA), suggesting coordinated osmolyte-antioxidant responses. Flavonoid content positively correlated with PPO activity, indicating synchronized phenolic metabolism and oxidation. ASA showed positive correlations with amino acids and antioxidant enzymes, suggesting integrated antioxidant responses. Conversely, APOX exhibited negative correlations with most parameters, particularly amino acids and proline. Oxidative stress markers (MDA, H_2_O_2_) showed negative correlations with antioxidant parameters (GSH, ASA), while GR demonstrated negative correlations with several parameters, indicating complex regulation of the glutathione-ascorbate cycle under stress conditions.

### 3.11 Transmission electron microscope analysis

**Figure 8:**
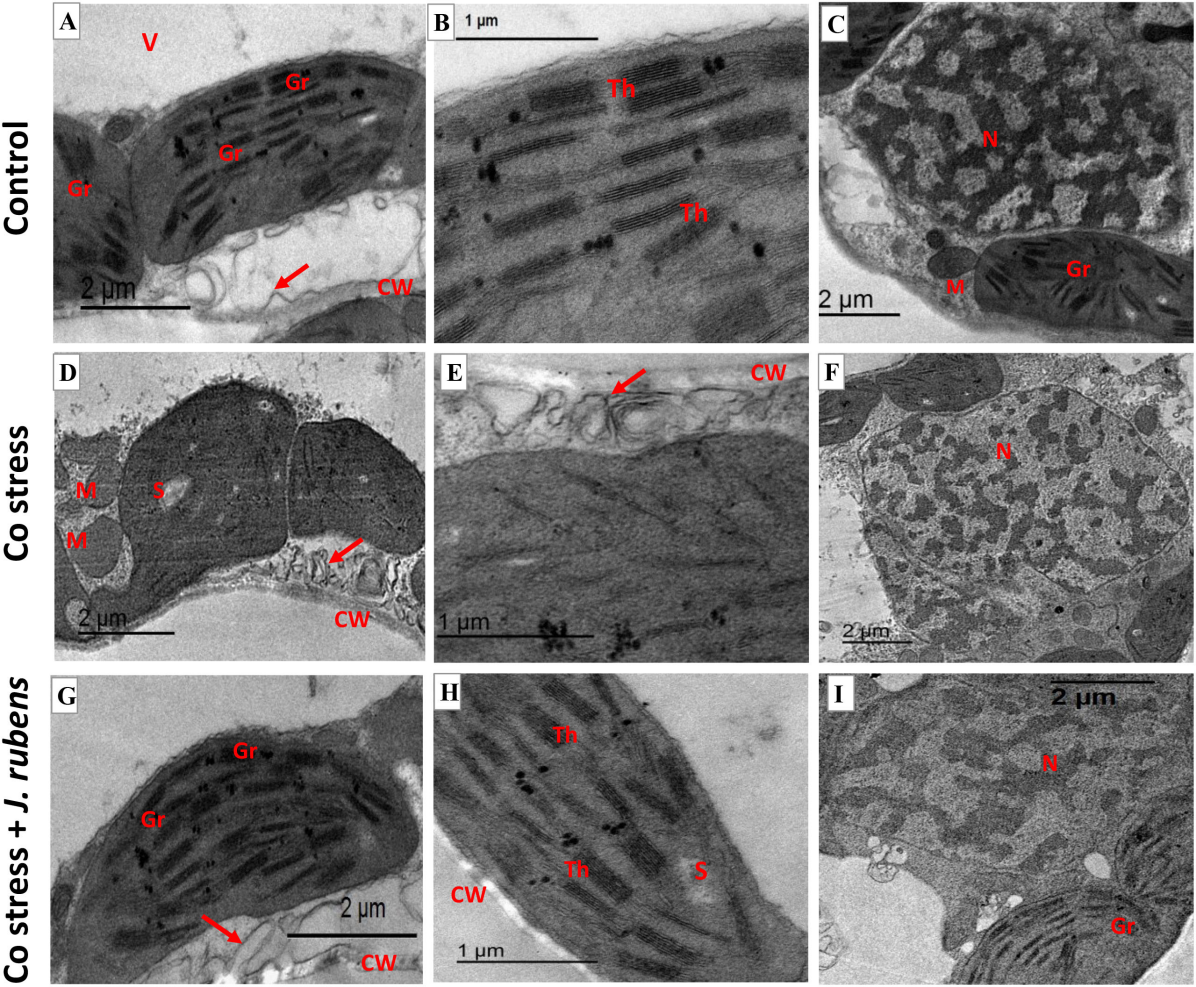
Transmission electron micrographs (TEM) of leaf mesophyll cells from wheat seedlings. **(A–C)** Untreated control, **(D–F)** exposed to 150 mM cobalt chloride, **(G–I)** primed with 1% *Jania rubens* extract prior to cobalt exposure. Samples were collected from the third fully expanded leaf after 10 days of treatment. Ultrastructural features include CW, cell wall; V, vacuole; Gr, grana; Th, thylakoids; S, starch; N, nucleus; M, mitochondria, and red arrows indicating cell wall microtubules.

The micrograph (**Figure 8**) depicting ultra-cellular structures demonstrated the modifications caused by Co stress in leaf mesophyll tissues. The ultrathin sections of the control (**Figures 8A-C**) exhibited well-developed cell organelles featuring a large central vacuole (**Figure 8A**) and systematically organized chloroplasts adjacent to the cell wall. Further, normal-oriented cell wall microtubules, well-developed chloroplasts having contact grana and regular arrangement of thylakoid membranes (**Figures 8A, B**), as well as a contact nucleus with distinct nuclear membrane (**Figure 8C**) were observed under control conditions. In contrast, Co stress-induced severe conformational changes such as swollen mitochondria and disorganized chloroplast shape (**Figure 7D**) with reduction or disappearance of thylakoid membranes (**Figures 8D, E**). Furthermore, it was evident that Co stress resulted in reorientation and disruption of cell wall microtubules (**Figures 6D, E**; Red arrow) and disintegrated nuclear membrane (**Figure 8F**). On the other hand, the cellular relieving capacity was maintained by *J. rubens* extract presoaking (**Figures 8G-I**) as exhibited via retaining the normal configuration of chloroplast, thylakoid membranes, nucleus, and cell wall microtubules (**Figure 8G**; Red arrow).

## 4 Discussion

Sustainable plant applications have become inevitable for the future of agriculture to face severe challenges of rising population, climate change, and scarcity of nutritional resources. Seaweed, including red, green, and brown marine algae, are now widely used as biostimulants to promote plant growth, productivity, and stress tolerance (Chiaiese et al., 2018; Hassan et al., 2021; Ashour et al., 2023). They possess phyto-eliciting properties, triggering plant defense mechanisms against pests, diseases, and environmental stresses such as drought, salinity, and heavy metal contamination (Ali et al., 2021). Recent literature has emphasized the unique bioactive properties of red seaweeds like *Jania rubens*, which contain diverse metabolites capable of modulating physiological responses in plants. According to Kumar et al. (2024), the application of red algae extracts triggers transcriptional reprogramming in treated crops, upregulating genes involved in nutrient uptake, stress response, and metabolic adjustment. Moreover, Hariharan et al. (2024) highlighted that seaweed-derived biostimulants not only enhance nutrient assimilation but also contribute to epigenetic modulation under metal and drought stress, enabling long-term resilience. These insights support the bioprotective potential of *Jania rubens*, reinforcing its relevance as a sustainable input in modern agronomic systems.

In this study, the red algae *J. rubens*, prevalent on the Egyptian Red Sea coast, was phytochemically analyzed for its beneficial effects on plants. Its high content of nutrients (K, N, Mg, Ca) and amino acids (such as aspartic acid, lysine, tyrosine, glutamic acid, and glycine) likely contribute to its positive effects. Additionally, *J. rubens* extract contains phytohormones like gibberellic acid, kinetin, and zeatin, which may help to chelate heavy metals and decrease their phytotoxicity (Sytar et al., 2013). The GC-MS analysis of *J. rubens* extract revealed a variety of fatty acids and fatty acid esters. Cis-vaccenic acid, the most abundant compound, and hexadecanoic acid, both may serve as potent antioxidants, combating oxidative damage (Kim et al., 2020; Qadir et al., 2020). Recent studies have indicated that fatty acids exogenous applications could induce plant defense responses via the expression of genes related to mitogen-activated protein kinase (MAPK) and jasmonic acid signaling pathways or help to mitigate oxidative damage (Kusumah et al., 2020; Seth et al., 2024). These pathways form part of an intricate signaling network involving calcium signaling, reactive oxygen species (ROS) perception, and hormonal cross-talk. As reviewed by Boyer et al. (2016), MAPK cascades serve as central hubs that integrate environmental cues, leading to the activation of transcription factors such as WRKY and MYB, which mediate stress-responsive gene expression. The presence of calcium-binding proteins in seaweed extracts, coupled with elevated Ca content in *J. rubens*, suggests that calcium-mediated signaling, including the activation of calmodulin and CDPKs, may play a role in the observed stress tolerance. This aligns with the work of Raja and Vidya (2023), who demonstrated that seaweed extract application modulated the expression of calcium-dependent kinase genes and stress-inducible antioxidant genes in rice exposed to metal toxicity. The wide array of compounds found in *J. rubens* extract, including phytohormones, fatty acids, amino acids, and minerals, could encourage their use in enhancing plant health or mitigating oxidative damage.

Cobalt as a metallic contaminant has been greatly released in agricultural systems as a direct consequence of excessive applications of fertilizers, livestock manures and compost, sewage sludge, and wastewater irrigation (Rashid et al., 2023). The irrigation with Co stress principally reduced growth traits of wheat seedlings including lengths, biomasses, and leaf area. The growth deterioration was accompanied by leaf chlorosis and stunted root thickening as signs of toxicity symptoms. The defined growth inhibitions can be explained by the decreased water content and mineral trafficking due to Co-induced cellular malfunctions. Several studies have documented cobalt’s toxicity to plant development and biomass accumulation in crops like barley and maize (Salam et al., 2022; Ali et al., 2025). Our current research indicates that cobalt’s inhibitory effects on root systems may be the primary cause of observed growth suppression. These root impacts restrict nutrient uptake, potentially explaining the growth inhibition we observed. The decrease in plant growth and biomass correlates directly with increased cobalt uptake and translocation throughout plant tissues. These negative effects are common responses to the oxidative damage induced by metal stress, resulting either from direct toxicity of accumulated metals or indirect limitations on nutrient and water uptake (Ragab and Saad-Allah, 2020a; Ragab et al., 2025).

Presoaking wheat seeds with *J. rubens* extract effectively restored normal growth traits and maintained cellular homeostasis under Co toxicity, as indicated by improved morphological characteristics and increased relative water content (RWC), suggesting enhanced water retention and osmotic balance. These findings confirm the bioprotective role of *J. rubens* extract in maintaining wheat growth under Co toxicity by promoting biomass accumulation and physiological resilience. The biostimulatory effects of seaweed extracts are primarily attributed to their content of phytohormones such as cytokinins, auxins, gibberellins, and essential hormones, which collectively promote plant growth and stress tolerance (Begum et al., 2018). Similar beneficial outcomes were reported by Dalal et al. (2019). where foliar application of *Kappaphycus alvarezii* extract enhanced root elongation, water retention, and metabolic performance in wheat varieties under salinity and drought stress. The high potassium content in *J. rubens* extract likely contributes to key physiological processes such as ion balance, leaf expansion, osmotic regulation, and energy translocation (Hasanuzzaman et al., 2018). Additionally, the abundant amino acids in *J. rubens* may enhance plant stress signaling and growth, acting as both metabolic precursors and signaling molecules, consistent with previous findings on amino acid-mediated stress mitigation (Abdel Latef et al., 2017).

Photosynthetic systems serve as reliable indicators of heavy metal toxicity in plant organisms (Ali et al., 2018; Fassela et al., 2020). Our investigation revealed that exposure to elevated cobalt concentrations significantly reduced multiple photosynthetic parameters, demonstrating the metal’s detrimental impact on these essential plant processes. With the cumulative decline in biomasses, the photosynthetic traits (Chl a, Chl b, and carotenoid levels) exhibited a sharp inhibition due to Co stress that appeared as acute chlorosis in wheat leaves (**Figure 1**). Cobalt stress is well-known for its detrimental effects on photosynthesis, leading to reduced plant growth and development (Salam et al., 2023). This decline in chlorophyll content may be attributed to Co’s interference with key steps in chlorophyll biosynthesis due to its high redox potential, as well as its inhibition of certain enzymes involved in chlorophyll production (Chandra and Kang, 2016). The recorded reduction in light-harvesting pigments could directly impact their photosynthetic efficiency and repress PSII activity (Fv/Fm). Further, Co-induced reduction in photosynthetic machinery is correlated to impaired plastid development and depleted Mg and Fe levels (**Figure 2**) which are essential metals involved in chlorophyll biosynthesis.

Application of *J. rubens* extract improved photosynthetic performance by reducing Co cellular toxicity that translated by enhanced morphophysiological traits and photosynthetic efficiency (**Figure 1**, **Table 4**). Similarly, El Boukhari et al. (2023) found that *Kappaphycus alvarezzi* red seaweed extract increased chlorophyll and carotenoid levels when applied to wheat varieties under salinity and drought stress. The increase in carotenoid content (**Table 4**) with *Jania* supplementation is likely due to its protective role in chloroplasts from photo-oxidative damage (Hashem et al., 2019; Saad-Allah and Ragab, 2020). Moreover, the significant K content in *J. rubens* extract can effectively improve photosynthetic enzyme activity, chlorophyll precursor production, and stomatal opening (El-Shenody et al., 2023) Thus, *Jania* application could supplement vital minerals including Mg, Fe, Ca, and K which could effectively improve photosynthesis.

Heavy metals are toxic to plants because they target crucial molecules and processes. Cobalt stress can interfere with mineral homeostasis, leading to changes in their accumulation in plant tissues. The unbalanced levels of K, Mg, and Fe due to Co toxicity. Cobalt accumulation was significantly higher in seedlings exposed to 150 mM CoCl_2_ compared to control plants. In roots, the Co content increased markedly, while shoots accumulated lower concentrations, interfering with vital physiological processes. The accumulation of K, Mg, and Fe in plant roots may be a common defense response to metal stress (Ghori et al., 2019) defining Co-induced root thickening to avoid metal toxicity and uptake. Accordingly, the decreased RWC and nutrient levels in shoot tissues may account for the harmed upper translocation and membrane-transport systems. The elevated Ca levels in both roots and shoots may achieve tolerance under Co stress, as calcium signaling is the crucial signaling component (Jalmi et al., 2018). Similarly, an increase in calcium content effectively mitigated ionic imbalances in *Triticum durum* under abiotic stress (Patel et al., 2018). Moreover, higher Co accumulation in wheat roots was recorded as compared to shoots, due to Co’s immobile behavior, aligning with previous findings that clarified higher Co retention in roots (Salam et al., 2024). The excessive Co levels, accumulated mainly in roots, can disrupt water and mineral uptake, block essential ions uptake, and interfere with normal cellular functions.

Seed priming with *J. rubens* extract pronouncedly relieved nutrient uptake impairment induced by Co stress and enhanced K, Mg, Fe, and Ca levels. On a similar scale, seaweed extracts considerably improved growth, photosynthetic pigments, and mineral contents in *Eruca vesicaria* (El-Shenody et al., 2023) and *Capsicum annuum* (Ashour et al., 2021). A particular upregulation of Ca contents in both roots and shoots was recorded compared to both stress and control treatments. In this regard, Ca abundance can improve the coordination of signaling networks involved in metal tolerance such as improving antioxidative responses, metal chelation, vacuole sequestration, and metal intake regulation through transporters coordinated by complex signaling networks (Jalmi et al., 2018).

Further, priming with *J. rubens* extract successfully reduced Co accumulation and uptake that may be associated with the synergetic roles of *Jania*-rich components. The seaweed priming advantages can induce the alertness phase through the activation of intrinsic and systemic plant responses, boosting plant resilience (Arioli et al., 2024). Despite being the first study to emphasize *J. rubens* extract’s capacity to reduce Co stress, it is evident that its rich nutritional composition is what sustains the plant’s mineral status.

Heavy metal stress often leads to overproduction of reactive oxygen species (ROS), such as H_2_O_2_ and hydroxyl radicals, which induce oxidative damage to lipids, proteins, and DNA. The accumulated Co ions, as redox-active metal, directly contribute to Haber-Weiss and Fenton processes, increasing the production of reactive oxygen species (ROS) and restricting metabolic pathways (Ragab and Saad-Allah, 2020b; Salam et al., 2024). Hence, exposure to Co stress caused acute oxidative stress and increased ROS generation, as evidenced by elevated H_2_O_2_, OH^•^, MDA, and EL (**Figure 3**). By increasing free radical levels, the Co-induced cellular toxicity triggers membrane lipid degradation and inhibits plant growth and development, leading to cell death (Ren et al., 2021). Despite the detected induction of PPO and CAT activities (**Figure 4**) in Co-treated leaves, H_2_O_2_ levels remained high, indicating these enzymes were insufficient to manage H_2_O_2_. Under extreme oxidative stress, an intense decrease in SOD, APOX, GPOX, and GR activities was reported (**Figure 4**), which compromised the plant’s antioxidant machinery and redox homeostasis (Salam et al., 2022), demonstrating Co detrimental effects. The enzyme deactivation is suggested to be a direct consequence of increased ROS levels or the interaction of metal ions with their functional cores (Abbas et al., 2018). Taken together, Co stress suppressed the enzymatic machinery of the ascorbate-glutathione scavenging cycle as a toxicity symptom that consequently accounts for the acceleration of O_2_^•−^, H_2_O_2,_ and OH^•^ accumulation.

Furthermore, the current study revealed depleted GSH content in response to Co stress, indicating its oxidation and exploitation as part of the ROS quenching process. Nevertheless, Co stress markedly inhibited most of the antioxidants; it improved the levels of ASA and total flavonoids. This enhancement could suggest an immediate defensive response to Co-induced homeostatic imbalance, along with detoxifying harmful ROS (Karuppanapandian and Kim, 2013). The correlation analysis showed that GSH and ASA (non-enzymatic antioxidants) show positive correlations with amino acids, indicating their role in stress protection. It can be concluded that wheat seedlings likely respond to Co toxicity via upregulation of non-enzymatic antioxidants, inducing ROS disproportionation. However, this was insufficient to restore homeostasis to its pre-stress level.

Applying *J. rubens* extract significantly reduced the production of free radicals and the contents of stress markers (H_2_O_2_, OH^•^, MDA, and electrolyte leakage) under Co stress. Similarly, the treatment with seaweed effectively enhanced ROS detoxification by regulating the expression of stress-responsive genes in soybean (Shukla et al., 2018) and reduced electrolyte leakage and MDA in wheat (Patel et al., 2018), alleviating abiotic stresses. It is worth noting that the application of *J. rubens* extract alone (without cobalt stress) resulted in the lowest levels of H_2_O_2_, OH•, and electrolyte leakage among all treatments, suggesting that the extract not only counteracts stress-induced oxidative damage but also improves the cellular redox status under normal conditions. These results collectively indicate that *J. rubens* extract contains potent bioactive compounds with significant antioxidant properties that effectively protect wheat seedlings against cobalt-induced oxidative stress.

Concerning antioxidant systems, soaking with *J. rubens* extract triggered a recovery response against Co-induced toxicity by enhancing the activities of SOD, APOX, GPOX, GR, and CAT. Likewise, the application of *J. rubens* extract was reported to improve stress tolerance in chickpeas by enhancing SOD and GPOX activities under high salinity (Abdel Latef et al., 2017). Compared to the NPK fertilizer treatments, *Capsicum annuum* treated with seaweed extract composed of *Ulva lactuca*, *Jania rubens* and *Pterocladiella capillacea* improved the total antioxidant activity of hot pepper by 1.17 folds (Ashour et al., 2021). The stimulation of Co-diminished SOD activity, as a response to *Jania* priming, represents a significant advantage as the primary mechanism of plant defense against ROS through O_2_^•−^ elimination. The stimulation of both APOX and GR could enhance the efficiency of the ascorbate-glutathione cycle as the major route of H_2_O_2_ scavenging (Asadi karam et al., 2017). The principal role of *J. rubens* extract in stress mitigation may be linked to its rich minerals, amino acids, and plant hormones contents. Hence, the treatment with *J. rubens* extract significantly induced antioxidant capacity in Co stressed seedlings via restricting metal toxicity and boosting the efficiency of ascorbate-glutathione cycle components. These results collectively indicate that *J. rubens* extract contains bioactive compounds capable of modulating the antioxidant defense system in wheat seedlings, enhancing their capacity to cope with cobalt-induced oxidative stress through the coordinated regulation of multiple antioxidant enzymes. This modulation appears to involve both the restoration of enzyme activities that are suppressed by cobalt stress (SOD, APOX, GPOX, GR) and the further enhancement of enzymes that are already stimulated by stress conditions (PPO, CAT).

Furthermore, *J. rubens* significantly increased the levels of antioxidant compounds including ASA, phenolics, and flavonoid contents. The most striking effect of *J. rubens* extract was a ~3-fold increase in flavonoid accumulation in both single treated and Co-stressed plants. Flavonoids, renowned for their free radical scavenging capabilities, likely play a central role in mitigating cobalt-induced oxidative stress. Flavonoid content shows interesting correlations with enzymatic antioxidants, particularly PPO, indicating the integration of non-enzymatic and enzymatic defense mechanisms. This flavonoid surge further supports the idea that *J. rubens* extract activates non-enzymatic antioxidant routes that reduce oxidative load more efficiently than traditional pathways.

Interestingly, a marked reduction in GSH levels was observed in plants treated with *J. rubens* extract, even under cobalt stress. This suggests a strategic metabolic shift in the antioxidant defense system, where phenolic and flavonoid-based mechanisms are favored over GSH-mediated pathways. This adaptive response is often linked to enhanced environmental stress tolerance in plants treated with complex biostimulants (Boutahiri et al., 2024). Moreover, these findings could endorse the effective exploitation of GSH in metal chelation and synthesis of peptides such as phytochelatins as a defensive mechanism. Under metal toxicity, the reduced GSH levels were linked to increased levels of high-affinity compounds with thiol-chelating groups (Sytar et al., 2013; Ragab and Saad-Allah, 2020b). Collectively, these findings highlight how *J. rubens* extract reprograms the non-enzymatic antioxidant network by enhancing phenolic and flavonoid biosynthesis while reducing dependence on GSH. This metabolic reconfiguration offers a refined and efficient strategy for combating heavy metal stress in wheat.

The exposure to Co stress resulted in a potent accumulation in proline and amino acids, which are mainly used for osmoregulation and compensating water deficits caused by heavy metals. These results were endorsed by the strong Pearson correlation (p<0.05) between amino acids and proline suggesting coordinated upregulation of osmolyte production, likely as a stress protection mechanism. Proline is known to chelate metal ions within plant cells and xylem sap, and it also acts as a cell wall plasticizer and an antioxidant by scavenging free radicals (Abbas et al., 2018). At the cellular level, certain amino acids act as osmolytes and signaling molecules to regulate ion transport (Sharma and Dietz, 2006). Amino acids mainly benefit plants by forming complexes with metal ions and transporting them into vacuoles, reducing metal toxicity (Alsafran et al., 2023). Despite their accumulation, proline and amino acids signal Co-induced oxidative injury rather than recovery, as noted by high ROS levels, physiological drought, membrane leakage, and lipid peroxidation.

Notably, pretreatment with *J. rubens* extract normalized proline and total amino acid concentrations to near-control levels in cobalt-stressed wheat plants. The normalization of amino acid levels in *J. rubens* extract-treated seedlings under cobalt stress indicates a restoration of normal nitrogen metabolism. This suggests that *Jania* preconditioning enhances metabolic efficiency, thereby reducing the reliance on classical stress-responsive osmolytes like proline. The extract’s rich amino acid and mineral composition likely underpins its exceptional biostimulant properties, as seaweed-derived amino acids are known to function as both metabolic intermediates and signaling molecules under abiotic stress (Hariharan et al., 2024). These findings collectively suggest that *J. rubens* extract contains bioactive compounds that effectively modulate nitrogen metabolism in wheat seedlings under cobalt stress, contributing to improved stress tolerance.

The current findings demonstrated that exposure to Co stress has detrimental impacts on cellular compartments. The TEM image revealed devastating ultrastructural changes related to chloroplast configuration. The disruption of thylakoid membranes and grana stacks was distinct, which limits the plant’s efficiency in capturing light energy and hinders photosynthetic machinery. The structural injury of chloroplasts and nuclear membrane coincided with the physiological toxicity of Co, including elevated ROS and distorted membrane permeability. Considering metal toxicity, the chloroplast was recorded to be the major target of ROS cellular damage due to the destruction of thylakoid membrane lipids (Kasim et al., 2014; Ragab et al., 2025). Similar severe damage was reported in subcellular compartments of Co-stressed maize plants (Salam et al., 2024). The acute conformational changes extended to cell wall strength and flexibility as an injury sign. The modifications of cell wall microtubules may impair the normal mechanical properties of a plant cell wall, leading to acute toxicity symptoms. The cellulose deposition of the cell wall is mainly governed by microtubules that principally maintain the cell’s mechanical integrity. Under stressful conditions, plants may modify the orientation and organization of microtubules to cope with the stress (Eleftheriou et al., 2016; Malivert et al., 2021). Further, the augmentation of free radicals may cause the breakdown of microtubules and other cytoskeletal elements, which is currently correlated to Co-inhibited plant growth.

Alternatively, the stimulating role of *J. rubens* extract was demonstrated by the structural restoration of cellular compartments (chloroplast, thylakoid membranes, nucleus, and cell wall). Priming with *Jania* primarily enhanced antioxidants and restricted ROS production, which mostly mitigated ultrastructure damage caused by Co.

The elevated Ca content due to *Jania* application could maintain the structural arrangement of cell wall microtubules. Besides its signaling role, Ca has an essential structural role in preserving cell wall integrity and stability, especially under stressful circumstances such as metal exposure (Parvin et al., 2019; Thor, 2019). Although the shortage of earlier information related to *J. rubens* extract and its role in alleviating metal cellular toxicity, its potential could be attributed to its stimulating properties.

## 5 Conclusion

This study demonstrates that cobalt (Co) exposure severely impairs wheat growth through morphophysiological disruptions, oxidative stress, and ultrastructural damage to key cellular components. Application of *Jania rubens* extract effectively mitigated these effects, enhancing plant resilience by stabilizing cellular structures, improving photosynthetic performance, and bolstering antioxidant defenses. These results highlight the potential of *J. rubens*-based biostimulants as a sustainable strategy for improving crop tolerance to heavy metal stress, particularly in contaminated agricultural systems. To advance practical applications, future research should prioritize large-scale field trials and in-depth molecular studies to validate efficacy under variable environmental conditions and further elucidate the mechanisms of stress mitigation.

## Data Availability

The original contributions presented in the study are included in the article/**Supplementary Material**. Further inquiries can be directed to the corresponding author.

## References

[B1] Abbas G. Murtaza B. Bibi I. Shahid M. Niazi N. K. Khan M. I.. (2018). Arsenic uptake, toxicity, detoxification, and speciation in plants: Physiological, biochemical, and molecular aspects. Int. J. Environ. Res. Public Health 15. doi: 10.3390/ijerph15010059 PMC5800158 29301332

[B2] Abdel Latef A. A. H. Srivastava A. K. Saber H. Alwaleed E. A. Tran L. S. P. (2017). Sargassum muticum and *Jania rubens* regulate amino acid metabolism to improve growth and alleviate salinity in chickpea. Sci. Rep. 7, 10537. doi: 10.1038/s41598-017-07692-w 28874670 PMC5585251

[B3] Akeel A. Jahan A. (2020). “Role of cobalt in plants: Its stress and alleviation,” in Contaminants in Agriculture: Sources, Impacts and Management. Springer, Cham, Switzerland. doi: 10.1007/978-3-030-41552-5_17

[B4] Aleem A. A. (1993). The marine algae of Alexandria. Egypt Alexandria 55, 1–139.

[B5] Ali S. Ali B. Sajid I. A. Ahmad S. Yousaf M. A. Ulhassan Z.. (2025). Synergistic effects of exogenous melatonin and zinc oxide nanoparticles in alleviating cobalt stress in Brassica napus: insights from stress-related markers and antioxidant machinery. Environ. Sci. Nano 12, 368–387. doi: 10.1039/D4EN00821A

[B6] Ali S. Gill R. A. Ulhassan Z. Najeeb U. Kanwar M. K. Abid M.. (2018). Insights on the responses of Brassica napus cultivars against the cobalt-stress as revealed by carbon assimilation, anatomical changes and secondary metabolites. Environ. Exp. Bot. 156, 183–196. doi: 10.1016/j.envexpbot.2018.07.004

[B7] Ali O. Ramsubhag A. Jayaraman J. (2021). Biostimulant properties of seaweed extracts in plants: Implications towards sustainable crop production. Plants 10. doi: 10.3390/plants10030531 PMC8000310 33808954

[B8] Allen S. Grimshaw H. M. Parkinson J. A. Quarmby C. (1989). Chemical analysis of ecological materials (London: Blackwell Scientific Publications).

[B9] Allen S. E. Grimshaw H. M. Parkinson J. A. Quarmby C. Roberts J. D. (1974). Chemical analysis of ecological materials (London: Blackwell Scientific Publications).

[B10] Alsafran M. Saleem M. H. Rizwan M. Al Jabri H. Usman K. Fahad S. (2023). An overview of heavy metals toxicity in plants, tolerance mechanism, and alleviation through lysine-chelation with micro-nutrients—A novel approach. Plant Growth Regul. 100, 337–354. doi: 10.1007/s10725-022-00940-8

[B11] Andersen M. E. (1985). Determination of glutathione and glutathione disulphide in biological samples. Methods Enzymol. 113, 548–555. doi: 10.1016/S0076-6879(85)13073-9 4088074

[B12] Anjum N. A. Singh H. P. Khan M. I. R. Masood A. Per T. S. Negi A.. (2015). Too much is bad—an appraisal of phytotoxicity of elevated plant-beneficial heavy metal ions. Environ. Sci. pollut. Res. 22, 3361–3382. doi: 10.1007/s11356-014-3849-9 25408077

[B13] Arioli T. Mattner S. W. Islam M. T. Tran T. L. C. Weisser M. Winberg P.. (2024). Applications of seaweed extracts in agriculture: An Australian perspective. J. Appl. Phycol. 36, 713–726. doi: 10.1007/s10811-023-03120-x

[B14] Arnon D. I. (1949). Copper enzymes in isolated chloroplasts. Polyphenoloxidase in Beta vulgaris. Plant Physiol. 24, 1. doi: 10.1104/pp.24.1.1 16654194 PMC437905

[B15] Asadi karam E. Keramat B. Sorbo S. Maresca V. Asrar Z. Mozafari H.. (2017). Interaction of triacontanol and arsenic on the ascorbate-glutathione cycle and their effects on the ultrastructure in Coriandrum sativum L. Environ. Exp. Bot. 141, 161–169. doi: 10.1016/j.envexpbot.2017.07.012

[B16] Ashour M. Al-Souti A. S. Hassan S. M. Ammar G. A. G. Goda A. M. A. S. El-Shenody R.. (2023). Commercial seaweed liquid extract as strawberry biostimulants and bioethanol production. Life 13, 85. doi: 10.3390/life13010085 PMC9865835 36676034

[B17] Ashour M. Hassan S. M. Elshobary M. E. Ammar G. A. G. Gaber A. Alsanie W. F.. (2021). Impact of commercial seaweed liquid extract (Tam^®^) biostimulant and its bioactive molecules on growth and antioxidant activities of hot pepper (Capsicum annuum). Plants 10, 1045. doi: 10.3390/plants10061045 34064289 PMC8224274

[B18] Bates L. S. Waldren R. P. Teare I. D. (1973). Rapid determination of free proline for water-stress studies. Plant Soil 39, 205–207. doi: 10.1007/BF00018060

[B19] Battah M. Mostfa M. Eladel H. Soror A. Tantawy M. (2021). Physiological Response of Fenugreek (Trigonella foenum-graecum L.) plant Treated by Farmyard Manure and Two Selected Seaweeds as Biofertilizers. Benha J. Appl. Sci. 6, 115–124. doi: 10.21608/bjas.2021.168294

[B20] Begum M. Bordoloi B. C. Singha D. D. Ojha N. J. (2018). Role of seaweed extract on growth, yield and quality of some agricultural crops: A review. Agric. Rev. 39, 321–326. doi: 10.18805/ag.r-1838

[B21] Beyer W. F. Jr. Fridovich I. (1987). Assaying for superoxide dismutase activity: some large consequences of minor changes in conditions. Anal. Biochem. 161, 559–566. doi: 10.1016/0003-2697(87)90489-1 3034103

[B22] Boutahiri S. Benrkia R. Tembeni B. Idowu O. E. Olatunji O. J. (2024). Effect of biostimulants on the chemical profile of food crops under normal and abiotic stress conditions. Curr. Plant Biol. 40, 100410. doi: 10.1016/j.cpb.2024.100410

[B23] Boyer R. L. Feng W. Gulbis N. Hajdu K. Harrison R. J. Jeffries P.. (2016). The use of arbuscular mycorrhizal fungi to improve strawberry production in coir substrate. Front. Plant Sci. 7. doi: 10.3389/fpls.2016.01237 PMC4991251 27594859

[B24] Chandra R. Kang H. (2016). Mixed heavy metal stress on photosynthesis, transpiration rate, and chlorophyll content in poplar hybrids. For. Sci. Technol. 12, 55–61. doi: 10.1080/21580103.2015.1044024

[B25] Chang C.-C. Yang M.-H. Wen H.-M. Chern J.-C. (2002). Estimation of total flavonoid content in propolis by two complementary colorimetric methods. J. Food Drug Anal. 10, 178–182. doi: 10.38212/2224-6614.2748

[B26] Chiaiese P. Corrado G. Colla G. Kyriacou M. C. Rouphael Y. (2018). Renewable sources of plant biostimulation: Microalgae as a sustainable means to improve crop performance. Front. Plant Sci. 871. doi: 10.3389/fpls.2018.01782 PMC6292864 30581447

[B27] Dalal A. Bourstein R. Haish N. Shenhar I. Wallach R. Moshelion M. (2019). Dynamic physiological phenotyping of drought-stressed pepper plants treated with “productivity-enhancing” and “survivability-enhancing” biostimulants. Front. Plant Sci. 10. doi: 10.3389/fpls.2019.00905 PMC6654182 31379898

[B28] Dixit D. Reddy C. R. K. (2017). Non-targeted secondary metabolite profile study for deciphering the cosmeceutical potential of red marine macro alga *Jania rubens*-An LCMS based approach. Cosmetics 4. doi: 10.3390/cosmetics4040045

[B29] Duan H. Liu W. Zhou L. Han B. Huo S. El-Sheekh M.. (2023). Improving saline-alkali soil and promoting wheat growth by co-applying potassium-solubilizing bacteria and cyanobacteria produced from brewery wastewater. Front. Environ. Sci. 11. doi: 10.3389/fenvs.2023.1170734

[B30] El Boukhari M. E. M. Barakate M. Drissi B. E. Bouhia Y. Lyamlouli K. (2023). Seaweed Extract Biostimulants Differentially act in Mitigating Drought Stress on Faba Bean (Vicia faba L.). J. Plant Growth Regul. 42, 5642–5652. doi: 10.1007/s00344-023-10945-w

[B31] Eleftheriou E. P. Adamakis I. D. S. Michalopoulou V. A. (2016). Hexavalent chromium-induced differential disruption of cortical microtubules in some Fabaceae species is correlated with acetylation of α-tubulin. Protoplasma 253, 531–542. doi: 10.1007/s00709-015-0831-4 26015161

[B32] El-Shenody R. A. Elshobary M. E. Ragab G. A. Huo S. Essa D. (2023). Towards biorefinery: Exploring the potential of seaweed-derived biodiesel and its residual biomass in improving the traits of Eruca vesicaria (L.) Cav. South Afr. J. Bot. 155, 361–371. doi: 10.1016/j.sajb.2023.02.029

[B33] Fassela P. Sinisha A. K. Brestic M. Puthur J. T. (2020). Special issue in honour of Prof. Reto J. Strasser - Chlorophyll a fluorescence parameters as indicators of a particular abiotic stress in rice. Photosynthetica 58, 293–300. doi: 10.32615/ps.2019.147

[B34] Ghori N. H. Ghori T. Hayat M. Q. Imadi S. R. Gul A. Altay V.. (2019). Heavy metal stress and responses in plants. Int. J. Environ. Sci. Technol. 16, 1807–1828. doi: 10.1007/s13762-019-02215-8

[B35] Grieve C. M. Grattan S. R. (1983). Rapid assay for determination of water soluble quaternary ammonium compounds. Plant Soil 70, 303–307. doi: 10.1007/BF02374789

[B36] Guiry M. D. Guiry G. M. (2019). AlgaeBase (Galway: World-wide electronic publication, National University of Ireland). Available online at: https://www.algaebase.org.

[B37] Halliwell B. Foyer C. H. (1978). Properties and physiological function of a glutathione reductase purified from spinach leaves by affinity chromatography. Planta 139, 9–17. doi: 10.1007/BF00390803 24414099

[B38] Hamed S. M. Abd El-Rhman A. A. Abdel-Raouf N. Ibraheem I. B. M. (2018). Role of marine macroalgae in plant protection & improvement for sustainable agriculture technology. Beni Suef. Univ. J. Basic Appl. Sci. 7, 104–110. doi: 10.1016/j.bjbas.2017.08.002

[B39] Hariharan G. Vathshalyan N. Galahitigama H. Wimalasiri U. Don Kapila Kumara G. (2024). Potential of foliar application of seaweed extracts as a biostimulant for abiotic stress alleviation on crop production. Rev. Agric. Sci. 12, 295–312. doi: 10.7831/ras.12.0_295

[B40] Hasanuzzaman M. Bhuyan M. Nahar K. Hossain Md. Mahmud J. Hossen Md.. (2018). Potassium: a vital regulator of plant responses and tolerance to abiotic stresses. Agronomy 8, 31. doi: 10.3390/agronomy8030031

[B41] Hashem H. A. Mansour H. A. El-Khawas S. A. Hassanein R. A. (2019). The potentiality of marine macro-algae as bio-fertilizers to improve the productivity and salt stress tolerance of canola (Brassica napus L.) plants. Agronomy 9. doi: 10.3390/agronomy9030146

[B42] Hassan S. H. Ashour M. Soliman A. A. F. Hassanien H. A. Alsanie W. F. Gaber A.. (2021). The potential of a new commercial seaweed extract in stimulating morphoagronomic and bioactive properties of Eruca vesicaria (L.) Cav. Sustianability 11, 1–21. doi: 10.3390/su13084485

[B43] Heath R. L. Packer L. (1968). Photoperoxidation in isolated chloroplasts: I. Kinetics and stoichiometry of fatty acid peroxidation. Arch. Biochem. Biophys. 125, 189–198. doi: 10.1016/0003-9861(68)90654-1 5655425

[B44] Horvath G. Kissimon J. Faludi-Dániel Á. (1972). Effect of light intensity on the formation of carotenoids in normal and mutant maize leaves. Phytochemistry 11, 183–187. doi: 10.1016/S0031-9422(00)89987-2

[B45] Hossain A. Skalicky M. Brestic M. Maitra S. Alam M. A. Syed M. A.. (2021). Consequences and mitigation strategies of abiotic stresses in wheat (Triticum aestivum l.) under the changing climate. Agronomy 11. doi: 10.3390/agronomy11020241

[B46] Hussein M. H. Eltanahy E. Al Bakry A. F. Elsafty N. Elshamy M. M. (2021). Seaweed extracts as prospective plant growth bio-stimulant and salinity stress alleviator for Vigna sinensis and Zea mays. J. Appl. Phycol. 33, 1273–1291. doi: 10.1007/s10811-020-02330-x

[B47] Ismail M. M. Ismail G. A. Elshobary M. E. (2023). Morpho-anatomical, and chemical characterization of some calcareous Mediterranean red algae species. Bot. Stud. 64, 10. doi: 10.1186/s40529-023-00373-0 37071314 PMC10113420

[B48] Jalmi S. K. Bhagat P. K. Verma D. Noryang S. Tayyeba S. Singh K.. (2018). Traversing the links between heavy metal stress and plant signaling. Front. Plant Sci. 9. doi: 10.3389/fpls.2018.00012 PMC5807407 29459874

[B49] Jindal K. K. Singh R. N. (1975). Phenolic content in male and female Carica papaya: a possible physiological marker for sex identification of vegetative seedlings. Physiol. Plant 33, 104–107. doi: 10.1111/j.1399-3054.1975.tb03774.x

[B50] Karuppanapandian T. Kim W. (2013). Cobalt-induced oxidative stress causes growth inhibition associated with enhanced lipid peroxidation and activates antioxidant responses in Indian mustard (Brassica juncea L.) leaves. Acta Physiol. Plant 35, 2429–2443. doi: 10.1007/s11738-013-1277-y

[B51] Kasim W. A. Abokassem E. M. Ragab G. A. Sewelam N. A. (2014). Alleviation of lead stress toxicity in Vigna unguiculata by salicylic acid. Egyptian J. Exp. Biol. 10, 37–49.

[B52] Kato M. Shimizu S. (1987). Chlorophyll metabolism in higher plants. VII. Chlorophyll degradation in senescing tobacco leaves; phenolic-dependent peroxidative degradation. Can. J. Bot. 65, 729–735. doi: 10.1139/b87-097

[B53] Kaur S. Singh H. P. Batish D. R. Negi A. Mahajan P. Rana S.. (2012). Arsenic (As) inhibits radicle emergence and elongation in Phaseolus aureus by altering starch-metabolizing enzymes vis-à-vis disruption of oxidative metabolism. Biol. Trace Elem. Res. 146, 360–368. doi: 10.1007/s12011-011-9258-8 22124861

[B54] Khairy H. M. El-Sheikh M. A. (2015). Antioxidant activity and mineral composition of three Mediterranean common seaweeds from Abu-Qir Bay, Egypt. Saudi J. Biol. Sci. 22, 623–630. doi: 10.1016/j.sjbs.2015.01.010 26288568 PMC4537878

[B55] Kim B. R. Kim H. M. Jin C. H. Kang S. Y. Kim J. B. Jeon Y. G.. (2020). Composition and antioxidant activities of volatile organic compounds in radiation-bred coreopsis cultivars. Plants 9. doi: 10.3390/plants9060717 PMC7356690 32512839

[B56] Kumar K. B. Khan P. A. (1982). Peroxidase and polyphenol oxidase in excised ragi (Eleusine corocana cv PR 202) leaves during senescence. Indian J. Exp. Biol. 20, 412–416. 7183525

[B57] Kumar G. Nanda S. Singh S. K. Kumar S. Singh D. Singh B. N.. (2024). Seaweed extracts: enhancing plant resilience to biotic and abiotic stresses. Front. Mar. Sci. 11. doi: 10.3389/fmars.2024.1457500

[B58] Kusumah D. Wakui M. Murakami M. Xie X. Yukihito K. Maeda I. (2020). Linoleic acid, α-linolenic acid, and monolinolenins as antibacterial substances in the heat-processed soybean fermented with Rhizopus oligosporus. Biosci. Biotechnol. Biochem. 84. doi: 10.1080/09168451.2020.1731299 32089087

[B59] Lee Y. P. Takahashi T. (1966). An improved colorimetric determination of amino acids with the use of ninhydrin. Anal. Biochem. 14, 71–77. doi: 10.1016/0003-2697(66)90057-1

[B60] Mahey S. Kumar R. Sharma M. Kumar V. Bhardwaj R. (2020). A critical review on toxicity of cobalt and its bioremediation strategies. SN Appl. Sci. 2. doi: 10.1007/s42452-020-3020-9

[B61] Malivert A. Erguvan Ö. Chevallier A. Dehem A. Friaud R. Liu M.. (2021). FERONIA and microtubules independently contribute to mechanical integrity in the Arabidopsis shoot. PloS Biol. 19. doi: 10.1371/journal.pbio.3001454 PMC8612563 34767544

[B62] Nakano Y. Asada K. (1981). Hydrogen peroxide is scavenged by ascorbate-specific peroxidase in spinach chloroplasts. Plant Cell Physiol. 22, 867–880. doi: 10.1093/oxfordjournals.pcp.a076232

[B63] Oser B. L. L. (1979). Hawk’s Physiological Chemistry. 14th Edn (New York: McGraw-Hills).

[B64] Parvin K. Nahar K. Hasanuzzaman M. Bhuyan M. H. M. B. Fujita M. (2019). “Calcium-mediated growth regulation and abiotic stress tolerance in plants,” in Plant Abiotic Stress Tolerance: Agronomic, Molecular and Biotechnological Approaches. Springer, Cham, Switzerland doi: 10.1007/978-3-030-06118-0_13

[B65] Passmore R. (1982). Nutritional Evaluation of Protein Foods Vol. 18. Eds. Pellett P. L. Young V. R. (Tokyo: United Nations University), 104–104. (1980), pp. 167. Exp Agric. doi: 10.1017/S0014479700013569

[B66] Patel K. Agarwal P. Agarwal P. K. (2018). Kappaphycus alvarezii sap mitigates abiotic-induced stress in Triticum durum by modulating metabolic coordination and improves growth and yield. J. Appl. Phycol. 30, 2659–2673. doi: 10.1007/s10811-018-1423-4

[B67] Qadir A. Aqil M. Ali A. Ahmad F. J. Ahmad S. Arif M.. (2020). GC-MS analysis of the methanolic extracts of Smilax China and Salix alba and their antioxidant activity. Turk. J. Chem. 44, 352–363. doi: 10.3906/KIM-1907-5 PMC7671229 33488162

[B68] Ragab G. A. Saad-Allah K. M. (2020a). Green synthesis of sulfur nanoparticles using Ocimum basilicum leaves and its prospective effect on manganese-stressed Helianthus annuus (L.) seedlings. Ecotoxicol. Environ. Saf. 191, 110242. doi: 10.1016/j.ecoenv.2020.110242 32004945

[B69] Ragab G. Saad-Allah K. (2020b). Seed priming with greenly synthesized sulfur nanoparticles enhances antioxidative defense machinery and restricts oxidative injury under manganese stress in Helianthus annuus (L.) seedlings. J. Plant Growth Regul. 5, 4091–4111. doi: 10.1007/s00344-020-10240-y

[B70] Ragab G. A. Saad-Allah K. M. Nessem A. A. (2025). Mitigating arsenate-induced phytotoxicity in fenugreek seedlings using garlic extract: insights into photosynthesis, arsenate uptake, antioxidative machinery and ultrastructure. J. Soil Sci. Plant Nutr. doi: 10.1007/s42729-025-02386-z

[B71] Raja B. Vidya R. (2023). Application of seaweed extracts to mitigate biotic and abiotic stresses in plants. Physiol. Mol. Biol. Plants 29, 641–661. doi: 10.1007/s12298-023-01313-9 37363418 PMC10284787

[B72] Rashid A. Schutte B. J. Ulery A. Deyholos M. K. Sanogo S. Lehnhoff E. A.. (2023). Heavy metal contamination in agricultural soil: environmental pollutants affecting crop health. Agronomy 13. doi: 10.3390/agronomy13061521

[B73] Ren L. Wang M. R. Wang Q. C. (2021). ROS-induced oxidative stress in plant cryopreservation: occurrence and alleviation. Planta 254. doi: 10.1007/s00425-021-03784-0 PMC8605965 34800184

[B74] Reynolds E. S. (1963). The use of lead citrate at high pH as an electron-opaque stain in electron microscopy. J. Cell Biol. 17, 208–212. doi: 10.1083/jcb.17.1.208 13986422 PMC2106263

[B75] Righini H. Roberti R. Cetrullo S. Flamigni F. Quintana A. M. Francioso O.. (2022). Jania adhaerens primes tomato seed against soil-borne pathogens. Horticulturae 8, 746. doi: 10.3390/horticulturae8080746

[B76] Saad-Allah K. M. Ragab G. A. (2020). Sulfur nanoparticles mediated improvement of salt tolerance in wheat relates to decreasing oxidative stress and regulating metabolic activity. Physiol. Mol. Biol. Plants 26, 2209–2223. doi: 10.1007/s12298-020-00899-8 33268924 PMC7688864

[B77] Sairam R. K. Srivastava G. C. Agarwal S. Meena R. C. (2005). Differences in antioxidant activity in response to salinity stress in tolerant and susceptible wheat genotypes. Biol. Plant 49, 85–91. doi: 10.1007/s10535-005-5091-2

[B78] Salam A. Afridi M. S. Khan A. R. Azhar W. Shuaiqi Y. Ulhassan Z.. (2023). “Cobalt induced toxicity and tolerance in plants: insights from omics approaches,” in Heavy Metal Toxicity and Tolerance in Plants: A Biological, Omics, and Genetic Engineering Approach, Wiley. Hoboken, New Jersey, USA 207–229.

[B79] Salam A. Khan A. R. Liu L. Yang S. Azhar W. Ulhassan Z.. (2022). Seed priming with zinc oxide nanoparticles downplayed ultrastructural damage and improved photosynthetic apparatus in maize under cobalt stress. J. Hazard. Mater. 423, 127021. doi: 10.1016/j.jhazmat.2021.127021 34488098

[B80] Salam A. Rehman M. Qi J. Khan A. R. Yang S. Zeeshan M.. (2024). Cobalt stress induces photosynthetic and ultrastructural distortion by disrupting cellular redox homeostasis in maize. Environ. Exp. Bot. 217, 105562. doi: 10.1016/j.envexpbot.2023.105562

[B81] Seth T. Asija S. Umar S. Gupta R. (2024). The intricate role of lipids in orchestrating plant defense responses. Plant Sci. 338. doi: 10.1016/j.plantsci.2023.111904 37925973

[B82] Sharma S. S. Dietz K. J. (2006). The significance of amino acids and amino acid-derived molecules in plant responses and adaptation to heavy metal stress. J. Exp. Bot. 57, 711–726. doi: 10.1093/jxb/erj073 16473893

[B83] Shindy W. W. Smith O. E. (1975). Identification of plant hormones from cotton ovules. Plant Physiol. 55, 550–554. doi: 10.1104/pp.55.3.550 16659120 PMC541656

[B84] Shukla P. S. Shotton K. Norman E. Neily W. Critchley A. T. Prithiviraj B. (2018). Seaweed extract improve drought tolerance of soybean by regulating stress-response genes. AoB Plants 10. doi: 10.1093/aobpla/plx051 PMC5751077 29308122

[B85] Sytar O. Kumar A. Latowski D. Kuczynska P. Strzałka K. Prasad M. N. V. (2013). Heavy metal-induced oxidative damage, defense reactions, and detoxification mechanisms in plants. Acta Physiol. Plant 35, 985–999. doi: 10.1007/s11738-012-1169-6

[B86] Thor K. (2019). Calcium—Nutrient and messenger. Front. Plant Sci. 10. doi: 10.3389/fpls.2019.00440 PMC6495005 31073302

[B87] Vasantharaja R. Abraham L. S. Inbakandan D. Thirugnanasambandam R. Senthilvelan T. Jabeen S. K. A.. (2019). Influence of seaweed extracts on growth, phytochemical contents and antioxidant capacity of cowpea (Vigna unguiculata L. Walp). Biocatal. Agric. Biotechnol. 17, 589–594. doi: 10.1016/j.bcab.2019.01.021

[B88] Velikova V. Yordanov I. Edreva A. (2000). Oxidative stress and some antioxidant systems in acid rain-treated bean plants: protective role of exogenous polyamines. Plant Sci. 151, 59–66. doi: 10.1016/S0168-9452(99)00197-1

[B89] Yadav S. L. Verma A. Nepalia V. (2016). Effect of phosphorus, sulphur and seaweed sap on growth, yield and nutrient uptake of chickpea (Cicer arietinum L.). Res. Crops 17, 496–502. doi: 10.5958/2348-7542.2016.00082.6

[B90] Zaborowska M. Kucharski J. Wyszkowska J. (2016). Biological activity of soil contaminated with cobalt, tin, and molybdenum. Environ. Monit. Assess. 18, 398. doi: 10.1007/s10661-016-5399-8 PMC4899498 27277093

