# Supplementary figures and images for: Unraveling the physiological and ultrastructural responses of wheat to combat cobalt stress and the protective role of Jania rubens related to antioxidant defense and cellular integrity
Source: Front Plant Sci. 2025 Jul 4;16:1621482. doi: 10.3389/fpls.2025.1621482 (PMC12271171; doi:10.3389/fpls.2025.1621482)

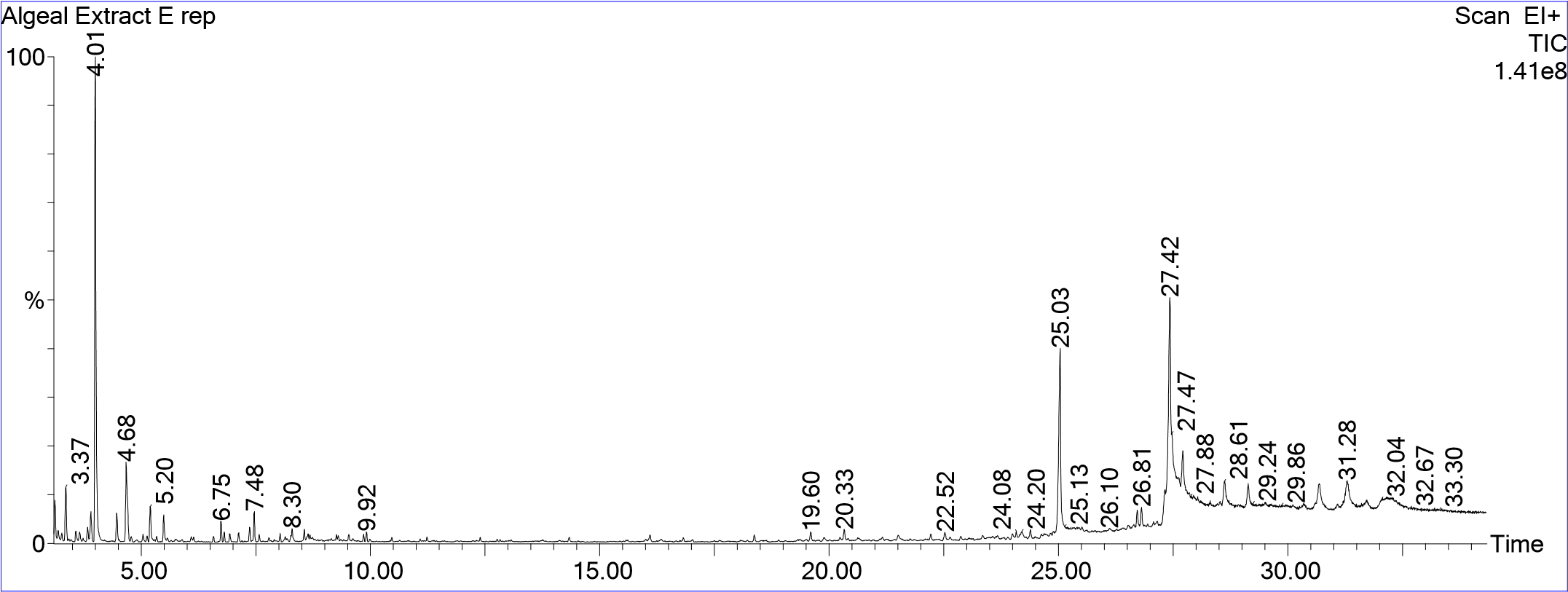


Fig. S1 Chromatogram for GC-MS of *J.* *rubens* extract

Supplement: Supplementary file 1 [file Table1.docx]
